# A distinctive ligand recognition mechanism by the human vasoactive intestinal polypeptide receptor 2

**DOI:** 10.1038/s41467-022-30041-z

**Published:** 2022-04-27

**Authors:** Yingna Xu, Wenbo Feng, Qingtong Zhou, Anyi Liang, Jie Li, Antao Dai, Fenghui Zhao, Jiahui Yan, Chuan-Wei Chen, Hao Li, Li-Hua Zhao, Tian Xia, Yi Jiang, H. Eric Xu, Dehua Yang, Ming-Wei Wang

**Affiliations:** 1 Department of Pharmacology, School of Basic Medical Sciences, Fudan University, Shanghai, 200032 China; 2 School of Artificial Intelligence and Automation, Huazhong University of Science and Technology, Wuhan, 430074 China; 3 The National Center for Drug Screening, Shanghai Institute of Materia Medica, Chinese Academy of Sciences, Shanghai, 201203 China; 4 The CAS Key Laboratory of Receptor Research, Shanghai Institute of Materia Medica, Chinese Academy of Sciences, Shanghai, 201203 China; 5 University of Chinese Academy of Sciences, Beijing, 100049 China; 6 Research Center for Deepsea Bioresources, Sanya, Hainan 572025 China; 7 School of Life Science and Technology, ShanghaiTech University, Shanghai, 201210 China; 8 Department of Chemistry, School of Science, The University of Tokyo, Tokyo, 113-0033 Japan

**Keywords:** Hormone receptors, Cryoelectron microscopy, G protein-coupled receptors

## Abstract

Class B1 of G protein-coupled receptors (GPCRs) comprises 15 members activated by physiologically important peptide hormones. Among them, vasoactive intestinal polypeptide receptor 2 (VIP2R) is expressed in the central and peripheral nervous systems and involved in a number of pathophysiological conditions, including pulmonary arterial hypertension, autoimmune and psychiatric disorders, in which it is thus a valuable drug target. Here, we report the cryo-electron microscopy structure of the human VIP2R bound to its endogenous ligand PACAP27 and the stimulatory G protein. Different from all reported peptide-bound class B1 GPCR structures, the N-terminal α-helix of VIP2R adopts a unique conformation that deeply inserts into a cleft between PACAP27 and the extracellular loop 1, thereby stabilizing the peptide-receptor interface. Its truncation or extension significantly decreased VIP2R-mediated cAMP accumulation. Our results provide additional information on peptide recognition and receptor activation among class B1 GPCRs and may facilitate the design of better therapeutics.

## Abstract (web summary)

Vasoactive intestinal polypeptide receptor 2 (VIP2R) is involved in immunity. Here, the authors report two cryo-EM structures of the VIP2R–G_s_ in complex with the endogenous peptide ligand PACAP27, revealing a unique interaction mode between PACAP27 and the receptor, stabilized by the N-terminal α-helix of VIP2R.

## Introduction

**Fig. 1:**
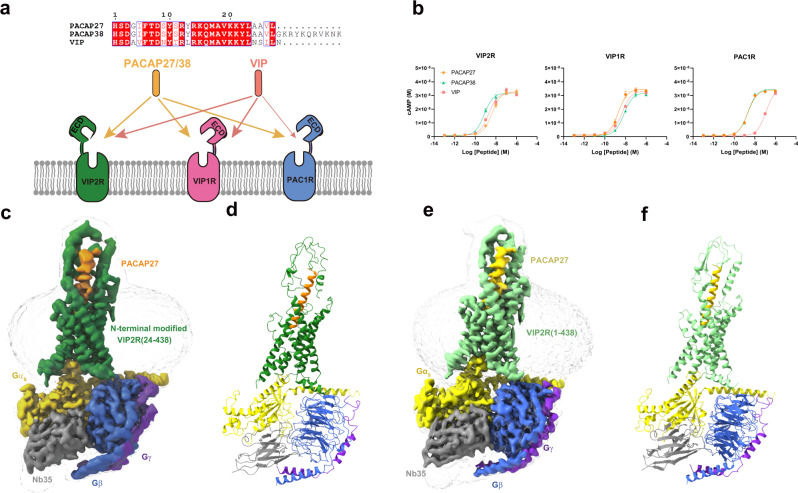
Cryo-EM structure of the PACAP27–VIP2R–G_s_ complex. **a** Binding specificity of PACAP and VIP receptor subfamily to the related peptide hormones. Sequence alignment of peptides is shown on the top panel. **b** Receptor signaling profiles of endogenous agonists PACAP27, PACAP38, and VIP. Data shown are means ± S.E.M. of at least three independent experiments performed in quadruplicate (*n* = 3–6). Source data are provided as a Source Data file. **c** Cut-through view of the cryo-EM density map illustrating the PACAP27–N-terminal modified VIP2R(24-438)–G_s_ complex and the disc-shaped micelle. **d** Model of the PACAP27–N-terminal modified VIP2R(24-438)–G_s_ complex as a cartoon, with PACAP27 as helix in orange. The receptor is shown in green, Gα_s_ in yellow, Gβ subunit in royal blue, Gγ subunit in violet, and Nb35 in gray. **e** Cut-through view of the cryo-EM density map illustrating the PACAP27–VIP2R(1-438)–G_s_ complex and the disc-shaped micelle. **f** Model of the PACAP27–VIP2R(1-438)–G_s_ complex as a cartoon, with PACAP27 as helix in gold. The receptor is shown in light green, Gα_s_ in yellow, Gβ subunit in royal blue, Gγ subunit in violet, and Nb35 in gray.

Vasoactive intestinal peptide (VIP) and pituitary adenylate cyclase-activating polypeptide (PACAP) are two important neuropeptides that exert a variety of physiological actions through three class B1 G protein-coupled receptors (GPCRs), namely PACAP type 1 receptor (PAC1R), VIP receptors 1 (VIP1R, or VPAC_1_) and 2 (VIP2R, or VPAC_2_)^1^. They share about 50% sequence similarities but mediate different functions such as neural development, calcium homeostasis, glucose metabolism, circadian rhythm, thermoregulation, inflammation, feeding behavior, pain, stress, and related endocrine responses^2–6^. Interestingly, PACAP (PACAP38 and PACAP27, a C-terminally truncated variant of PACAP38) and VIP have comparable affinity at VIP1R and VIP2R, but PACAP is 50 to 400-fold more potent than VIP at the PAC1R (Fig. 1a, b). Extensively expressed in the central and peripheral nervous systems^7,8^, VIP2R is involved in a number of pathophysiological conditions, showing a great potential as a therapeutic target for pulmonary arterial hypertension, chronic obstructive pulmonary disease (COPD), cancer, asthma, autoimmune and psychiatric disorders^9–12^.

A comprehensive molecular understanding of VIP/PACAP recognition and receptor activation is important to the design of better drug candidates. Further to the recently reported cryogenic electron microscopy (cryo-EM) structures of VIP1R and PAC1R^13–16^, here we determine two single-particle cryo-EM structures of the human VIP2R in complex with PACAP27 and the stimulatory G protein (G_s_), at global resolutions of 3.4 and 2.7 Å, respectively. Combined with molecular dynamics (MD) simulation and functional studies, these structures provide valuable insights into a distinctive molecular mechanism governing ligand recognition and VIP2R activation.

## Results

### Structure determination

To prepare a high-quality human VIP2R–G_s_ complex, we initially added a double tag of maltose-binding protein (MBP) at the C terminus, replaced the native signal peptide at the N terminus with the prolactin precursor sequence followed by two introduced tags (Flag and 8×His tag), and employed the NanoBiT tethering strategy (Supplementary Fig. 1a–f)^13,17–20^. Considering that such N-terminal modification reduced both the production of cAMP and the ligand-binding ability of our VIP2R construct (Supplementary Fig. 1j, k), we built a complete wild-type (WT) construct VIP2R(1-438) without any tags and retained the native signal peptide (Supplementary Fig. 1b, g–m). After sample preparation, cryo-EM data collection and analysis (Supplementary Figs. 1–3), 3D consensus density maps were reconstructed with a global resolution of 3.4 Å for the PACAP27–N-terminal modified VIP2R(24-438)–G_s_ and 2.7 Å for the PACAP27–VIP2R(1-438)–G_s_ complexes, respectively (Fig. 1c–f, Supplementary Fig. 2 and Supplementary Table 1). The cryo-EM maps allowed us to build an accurate model for most regions of the complex except for the flexible α-helical domain (AHD) of Gα_s_ and the VIP2R residues from P313 to S321 in the intracellular loop 3 (ICL3) (Supplementary Fig. 3). The extracellular domain (ECD) had a lower resolution due to the high conformational flexibility widely observed among class B1 GPCRs^21–23^ and therefore was modeled on the rigid-body fitted crystal structure of VIP2R ECD (PDB code: 2X57). The density of the LgBiT is invisible in our cryo-EM maps due to its relative flexibility, like many other GPCR–G protein complex structures determined using the NanoBiT tethering strategy^13,17–20^. Considering the higher resolution feature and native-like signaling profile of the PACAP27–VIP2R(1-438)–G_s_ complex, structural analysis of VIP2R was performed based on this model.

### Overall structure

The PACAP27–VIP2R–G_s_ complex adopts a typical architecture of the activated class B1 GPCR conformations, characterized by a single straight helix of PACAP27 that interacts with both ECD and the transmembrane domain (TMD), a sharp kink in the middle of the TM helix 6 (TM6) thereby opening the cytoplasmic face, and the insertion of the C-terminal α5 helix of the Gα_s_ into the receptor core (Fig. 1d, f). Its overall structure is similar to other class B1 GPCRs–G_s_ complexes^24–27^. For the PACAP and VIP receptor subfamily, the structure of PACAP27-bound VIP2R displays a high degree of resemblance compared to previously reported structures with Cα root mean square deviation (RMSD) values ranging from 0.70 to 1.04 Å (PDB codes: 6LPB, 6M1H, 6M1I, 6P9Y, and 6VN7)^13–16^.

**Fig. 2:**
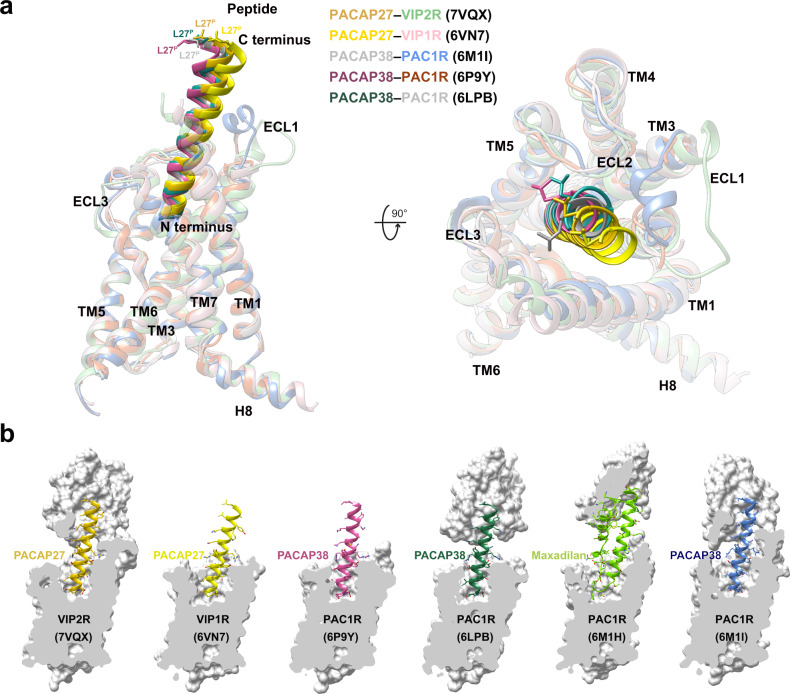
Structural comparison of active VIP2R, VIP1R, and PAC1R. **a** Superimposition of PACAP27–VIP2R, PACAP27–VIP1R (PDB code: 6VN7)^13^ and PACAP38–PAC1R (PDB codes: 6M1I, 6LPB, and 6P9Y)^14–16^ reveals a high structural similarity. Receptor ECD and G protein are omitted for clarity. **b** Surface representations of peptide-binding pockets among VIP2R, VIP1R, and PAC1R. The bound peptides are shown as sticks ribbon and sticks.

As shown in Fig. 2, the N termini of the bound PACAP27 and PACAP38 overlapped well and penetrated into the TMD core by an almost identical angle and orientation, exhibiting a shared ligand recognition pattern (Supplementary Table 2). Notable differences in both position and orientation at the peptide C-terminal halves were observed via the surrounding ECD, extracellular loop 1 (ECL1), and the extracellular tip of TM1 conformations that are unique to VIP2R. Specifically, the VIP2R-bound PACAP27 was rotated by 4.6° compared to that in the complex with VIP1R; such a movement shifted its C terminus toward the TMD core by 4.2 Å (measured by the Cα of L27^P^, P indicates that the residue belongs to the peptide). By choosing a more relaxed ECL1 conformation rather than the ordered two-turn α-helix found in the ECL1 of PAC1R (Fig. 2a), VIP2R reduced the contacts between ECL1 and peptide evidenced by a decrease in the buried surface area from 282 Å^2^ (PAC1R) to 228 Å^2^ (VIP2R). Consequently, the C terminus of PACAP27 bound by VIP2R moved toward ECL1 by 3.2 Å in comparison with that of PACAP38 bound by PAC1R (measured by the Cα of L27^P^). Collectively, these common and unique structural features highlight the complexity of peptide recognition among VIP2R, VIP1R, and PAC1R.

### Ligand recognition

**Fig. 3:**
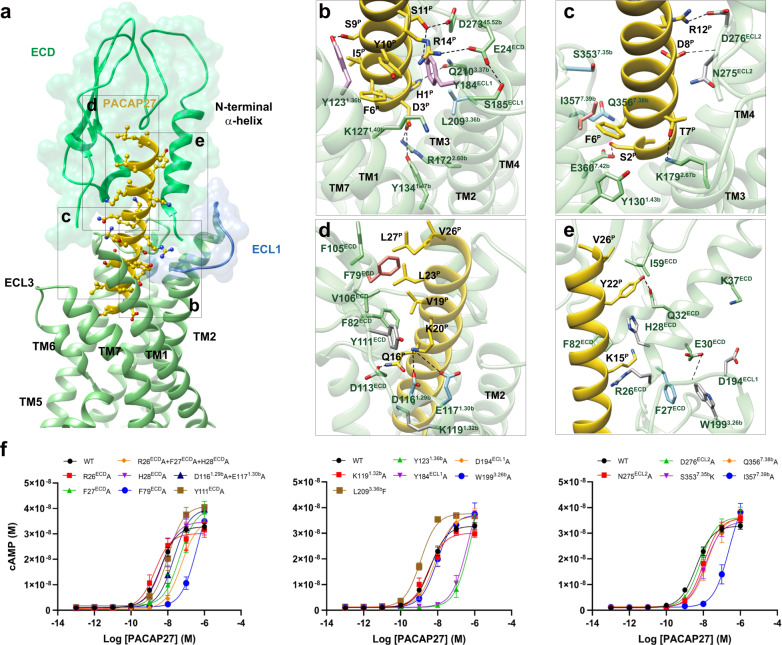
Molecular recognition of PACAP27 by VIP2R. **a** The binding mode of PACAP27 (gold) with VIP2R (light green), showing that the N-terminal half of PACAP27 penetrates into a pocket formed by TMs 1-3, TMs 5-7, and ECLs 1-3, whereas the C-terminal half is recognized by the ECD, ECL1, and TM1. The ECD and ECL1 are shown as surface. **b**–**e** Close-up views of the interactions between PACAP27 and VIP2R. Key residues are shown as sticks. Concentration-response curves of VIP2R mutants that show <3-fold effect (dark gray), 3- to 15-fold effect (sky blue), 15- to 100-fold effect (salmon), or >100-fold effect (plum). **f** Signaling profiles of VIP2R mutants. cAMP accumulation in wild-type (WT) and single-point mutated VIP2R expressing CHO-K1 cells. Signals were normalized to the maximum response of the WT and concentration-response curves were analyzed using a three-parameter logistic equation. All data were generated and graphed as means ± S.E.M. of at least three independent experiments, conducted in quadruplicate (*n* = 3–6). Source data are provided as a Source Data file.

The active VIP2R structure shows that PACAP27 is stably anchored through two interaction networks (Fig. 3a): the first is comprised of the peptide N-terminal half (residues 1–13) and the residues in the lower half of the ligand-binding pocket (Fig. 3b, c), while the second connects the peptide C-terminal half with the ECD (especially the N-terminal α-helix), ECL1 and the stalk region (Fig. 3d, e).

In the first network, H1^P^, D3^P^, I5^P^, F6^P^, D8^P^, S9^P^, Y10^P^, and S11^P^ contribute to common interactions with the conserved residues among VIP2R, VIP1R, and PAC1R, while S2^P^, G4^P^, R12^P^, and Y13^P^ make receptor-specific interactions. For common interactions, H1^P^ is oriented toward TM3 with the formation of a hydrogen bond with Q210^3.37b^ (class B1 GPCR numbering in superscript), similar interaction is also observed in VIP1R and PAC1R. D3^P^ is highly conserved in class B1 GPCR peptide hormones, which simultaneously forms one salt bridge with R172^2.60b^ and one hydrogen bond with Y134^1.47b^ in the cases of VIP2R, VIP1R, and PAC1R. Polar interactions also occurred between D8^P^ and N275^ECL2^ (backbone nitrogen), S9^P^ and Y123^1.36b^ (side-chain oxygen), as well as S11^P^ and Y184^ECL1^ (side-chain oxygen)/D273^45.52b^ (side-chain oxygen). I5^P^, F6^P^, and Y10^P^ contribute massive nonpolar interactions with the conserved residues in TM1 and TM7 including Y123^1.36b^, K127^1.40b^, Y130^1.43b^, I357^7.39b^, and L361^7.43b^. Consistently, the substitution of the residue at 1.36b by alanine greatly reduced PACAP27 potency for three receptors (155-fold for VIP2R, 108-fold for VIP1R, and 39-fold for PAC1R) (Fig. 3f, Supplementary Table 3). The VIP2R mutant I357^7.39b^A significantly diminished the PACAP27 potency by 64-fold (Fig. 3f), while equivalent mutations in VIP1R (M370^7.39b^A) and PAC1R (L382^7.39b^A) only displayed moderate effects (8-fold for VIP1R and 10-fold for PAC1R) (Supplementary Table 3). Besides the above common interactions among VIP2R, VIP1R, and PAC1R, distinct amino acids in the equivalent positions of these three receptors fine tune the specific ligand-receptor recognition pattern. S2^P^ forms one hydrogen bond with E360^7.42b^ of VIP2R, such an interaction is neither found in VIP1R (K369^7.38b^) nor in PAC1R (R381^7.38b^). By using leucine at position 3.36b instead of phenylalanine in both VIP1R and PAC1R, the contact between G4^P^ and VIP2R is slightly reduced. Consistently, the VIP2R mutant L209^3.36b^F increased the potency of PACAP27 by 3-fold (Fig. 3f). A similar phenomenon was observed for Y13^P^, where T136^1.33b^ in VIP1R and Y148^1.34b^ in PAC1R additionally provide one hydrogen bond and one stacking interaction, respectively. However, R12^P^ forms a salt bridge with D276 in the ECL2 of VIP2R (Fig. 3c), which is not observed in VIP1R, mainly due to the lack of negatively charged residues caused by a shorter ECL2 (by one amino acid compared to VIP2R or PAC1R).

The second network stabilizes the peptide–ECD–ECL1–TM1 interface through massive nonpolar and polar interactions. The C terminus of PACAP27 occupies a complementary binding groove of the ECD, consisting of a series of hydrophobic residues (I59^ECD^, F79^ECD^, F105^ECD^, and Y111^ECD^) that make extensive hydrophobic contacts with PACAP27 via V19^P^, F22^P^, L23^P^, V26^P^, and L27^P^, consistent with that seen in other class B1 GPCRs such as GLP-1R^24^, GHRHR^18^, and PAC1R^14^. For the polar contacts, R14^P^ forms one salt bridge with E24^ECD^ and stacking interactions with Y184^ECL1^, the side-chain of Q16^P^ extends to the stalk with the formation of one hydrogen bond with D113^ECD^, while K20^P^ points to two adjacent negatively charged residues (D116^1.29b^ and E117^1.30b^) in the extracellular tip of TM1 (Fig. 3b, d). These observations received the support of our mutagenesis studies, where mutant F79^ECD^A, and Y184^ECL1^A decreased the potency of PACAP27-induced cAMP signaling by 96-fold, and 104-fold, respectively (Fig. 3f).

**Fig. 4:**
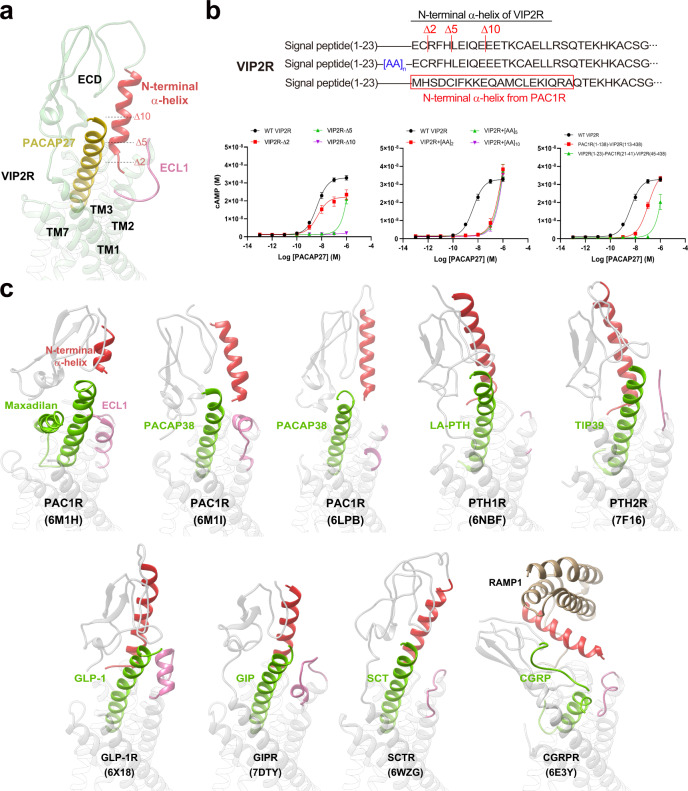
Unique conformation of the N-terminal α-helix of VIP2R in class B1 GPCRs. **a** Close-up view of the PACAP27–ECD–ECL1 interface shows that the N-terminal α-helix filled the cleft between PACAP27 and ECL1, thereby stabilizing the complex. **b** Signaling profiles of VIP2R with truncated or extended ECD and substitution of the N-terminal α-helix or ECD from PAC1R in response to PACAP27. Data are presented as means ± S.E.M. of at least three independent experiments (*n* = 3–6). Source data are provided as a Source Data file. WT, wild-type. Δ, residue truncation. [AA]_2_, extend the receptor N terminus by two amino acids (GS); [AA]_5_, five amino acids (GSSGG); [AA]_10_, ten amino acids (GSSGGGGSGG). **c** Conformational comparison of the N-terminal α-helix among peptide-bound class B1 GPCR structures. All structures are superimposed on the GLP-1-bound GLP-1R (PDB code: 6X18)^24^ using the Cα carbons of the TMD residues. The receptor is shown in gray, peptide in green, ECL1 in pink, and the N-terminal α-helix in red.

The most profound structural feature resides in the upper half of the PACAP27-bound VIP2R showing a position and orientation of the ECD N-terminal α-helix distinctive from all available class B1 GPCR structures reported to date (Figs. 3 and 4). Specifically, the tip of the N-terminal α-helix moved down toward the TMD by 13.8 Å relative to that of PAC1R (measured by the Cα of E24 in VIP2R and D23 in PAC1R) and inserted into a cleft between PACAP27 and the ECL1 (Fig. 4a). Such a unique conformation was probably caused by an outward movement of the ECL1, which appears to be conformationally more flexible as it is longer by three amino acids with the presence of two proline residues (P193^ECL1^ and P196^ECL1^) compared to the ECL1 of PAC1R (Fig. 4c). The inserted N-terminal α-helix stabilizes the peptide and ECL1 conformations via multiple contacts including two salt bridges (R14^P^ and E24^ECD^, K37^ECD^ and D194^ECL1^), two hydrogen bonds (E24^ECD^ and S185^ECL1^, E30^ECD^ and C192^ECL1^) and several hydrophobic contacts (R14^P^ and C25^ECD^, K15^P^ and H28^ECD^, Y22^P^ and Q32^ECD^, F27^ECD^ and W199^3.26b^) (Fig. 3b, e). Consistently, MD simulations found that the unwound N-terminal α-helix of the ECD could stably insert into the cleft between PACAP27 and ECL1 through its tip residues (D24^ECD^ to I31^ECD^), as supported by both the interface area and representative minimum distances (E24^ECD^-R14^P^ and F27^ECD^-W199^3.26b^) (Supplementary Fig. 4a–c). Consequently, PACAP27 was greatly stabilized to successfully maintain these strong polar contacts with the TMD and ECD residues during MD simulations, thereby utilizing a similar interface area as the cryo-EM structure (Supplementary Fig. 4d–g).

To reveal the functional roles of the N-terminal α-helix in the presence of PACAP27, we truncated the N-terminal α-helix in a systemic manner and measured cAMP responses subsequently (Fig. 4b). For VIP2R, cAMP signaling was completely abolished when five or more residues were truncated (Fig. 4b and Supplementary Table 5). In contrast, the action of both VIP1R and PAC1R do not require the participation of the N-terminal α-helix or even the ECD, whose maximal responses of receptor-mediated cAMP accumulation in the presence of PACAP27 were retained when 5 or 10 residues, or even the entire ECD were truncated (Supplementary Table 5). A similar phenomenon was observed in concentration-response characteristics of the N-terminal α-helix extension. The addition of a flexible linker (G/S) at the receptor N terminus had neglectable effects on ligand binding and receptor activation for both VIP1R and PAC1R (Supplementary Table 5). However, VIP2R was extremely sensitive to the N-terminal α-helix extension, where two, five, or ten introduced amino acids (G/S) reduced PACAP27 potency by 201-fold, 343-fold, and 471-fold, respectively (Fig. 4b), indicating a curial role of the N-terminal α-helix length in VIP2R functioning. Interestingly, the substitution of the N-terminal α-helix or whole ECD of VIP2R by corresponding regions of PAC1R completely abolished or greatly reduced PACAP potency (by 25.6-fold), respectively (Fig. 4b and Supplementary Table 5). Besides, triple mutation at the beginning of the N-terminal α-helix (R26^ECD^A + F27^ECD^A + H28^ECD^A) increased the EC_50_ of VIP2R-mediated cAMP accumulation by 13.3-fold, relative to the single-point mutated constructs (EC_50_ decreased by 2.1-fold for R26^ECD^A, increased by 10.6- and 1.2-fold for F27^ECD^A and H28^ECD^A, respectively) (Fig. 3f and Supplementary Table 3). Collectively, our results suggest that VIP2R possesses a distinct molecular mechanism for peptide recognition and receptor activation.

Inspired by such a unique N-terminal α-helix conformation of VIP2R, we performed structural analysis across class B1 GPCRs (Fig. 4c). Despite a high sequence similarity to VIP2R, PAC1R pulls out its N-terminal α-helix away from the peptide-ECL1 cleft to make negligible contact with either peptide or ECL1^14^. Alternatively, compared to the PACAP38-bound conformation, PAC1R adjusts its ECD to move upward, while ECL1, TM1, and TM2 shift outward for maxadilan (a native peptide from the sand fly) recognition, indicative of a high structural adaptability of PAC1R. In the case of parathyroid hormone (PTH) receptors whose ECL1s are unstructured, PTH1R^21^ and PTH2R^26^ rotate their N-terminal α-helices to stand upwards in line with the bound peptides, thereby providing additional contacts with the latter. Different from the ECL1-top conformation seen in PAC1R or the ECL2-top position observed in PTH1R/PTH2R, the N-terminal α-helices of glucagon receptor family members (GLP-1R^24^, GIPR^25^, and SCTR^28^ shown in Fig. 4c) locate in the middle of ECL1 and ECL2, and stabilize the peptide C terminus with the assistance of ECL1. As for calcitonin gene-related peptide receptor (CGRPR)^28^, the N-terminal α-helix rotates downward to cover the orthosteric site, probably due to the loop conformation in the C-terminal region of GCRP, as well as a shorter ECL1 compared to VIP2R or GLP-1R. Taken together, these observations demonstrate the diversity and flexibility of N-terminal α-helices among class B1 GPCRs, and highlight the importance of interplay among N-terminal α-helix, ECL1, and peptide.

### G protein coupling

**Fig. 5:**
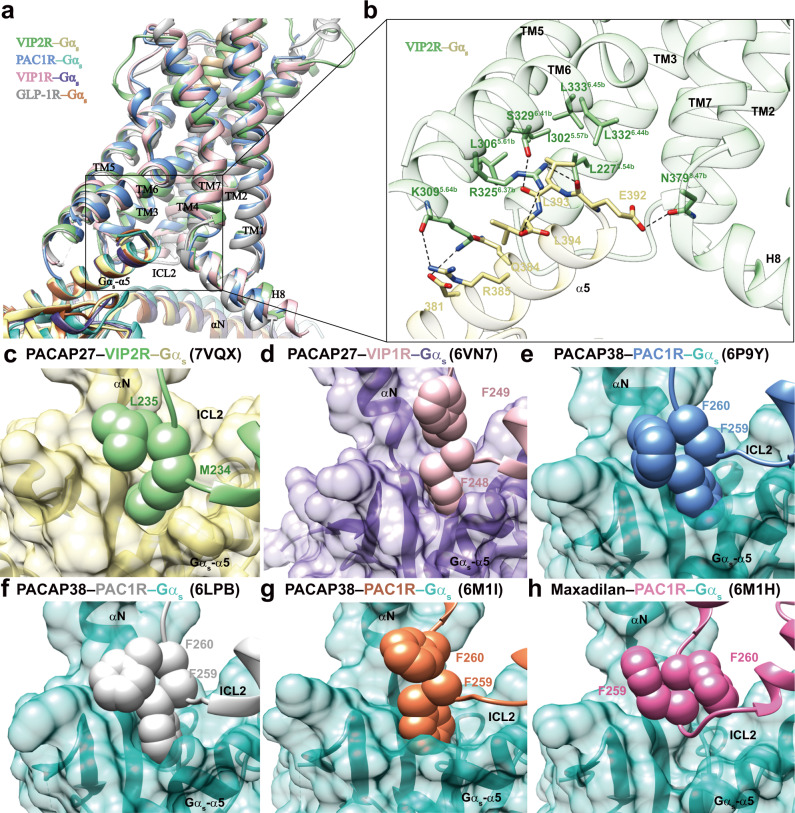
G protein coupling of VIP2R. **a** Comparison of G protein coupling among VIP2R (PDB code: 7VQX), VIP1R (PDB code: 6VN7), PAC1R (PDB code: 6P9Y)^16^ and GLP-1R (PDB code: 6X18)^24^. The receptors and G proteins are colored as the labels. **b** Interaction between VIP2R and the C terminus of Gα_s_. **c**–**h** Comparison of the interactions between ICL2 and Gα_s_ for VIP2R (PDB code: 7VQX), VIP1R (PDB code: 6VN7), and PAC1R (PDB codes: 6P9Y, 6LPB, 6M1H, and 6M1I)^14–16^. Residues involved in the interactions are shown as sphere and Gα_s_ is shown as surface.

Comparison of the VIP2R–G_s_ complex with other class B1 GPCRs suggests that VIP2R follows common receptor activation and G protein coupling mechanisms shared by class B1 GPCRs in terms of TM and residue level features^14,18,24,29^. The former is characteristic of an outward movement of the intracellular portion of TM6 and the bend of the extracellular half of TM7 towards TM6. The latter involves rearrangement of the central polar network, HETX motif (H^2.50b^, E^3.50b^, T^6.42b^, and Y^7.57b^), and TM2-6-7-helix 8 polar network^14,18,24,29^. Looking at the G protein coupling, G_s_ protein is anchored by the α5 helix of Gα_s_ (GαH5), thereby fitting to the cytoplasmic cavity made by TMs 2, 3, 5, 6, 7, and ICLs 1-3 of VIP2R with the formation of multiple polar and massive hydrophobic interactions (Fig. 5a, b). There are some receptor-specific structural features displayed by the ICL2 (Fig. 5c–h). Different from the benzene ring of F248^ICL2^ (VIP1R) and F259^ICL2^ (PAC1R) that inserted into a hydrophobic pocket of Gα_s_, M234^ICL2^ of VIP2R makes slightly reduced interactions with Gα_s_ (Fig. 5c). However, the dipped down side-chain conformation of L235^ICL2^ provides additional hydrophobic contacts to stabilize the VIP2R–G_s_ interface, while that of VIP1R (F249^ICL2^) rotates away from the interface that has negligible contact with G protein (Fig. 5d). For PAC1R, the notable structural variance was observed in the side-chain orientation of F260^ICL2^ (Fig. 5e–h), indicating the dynamic nature of ICL2 in receptor-G protein coupling.

## Discussion

The cryo-EM structure of the PACAP27–VIP2R–G_s_ complex presented here reveals a distinctive and previously unknown peptide recognition mechanism responsible for ligand specificity at near-atomic resolution. Combined with functional and MD simulation studies, we determined that PACAP27 is recognized by VIP2R through its N-terminal α-helix that inserts into the cleft between the peptide and the ECL1. Such a phenomenon was only observed in VIP2R, not in closely related VIP1R and PAC1R, indicating a diversified receptor responsiveness to the same ligand. The importance of receptor N terminus was also elegantly demonstrated in a recent study on compound 2-bound GLP-1R showing another TMD-interacted conformation for the N-terminal α-helix, which penetrates to the GLP-1 binding site and activates the receptor^19^. The interplay between the N-terminal α-helix and TMD observed in different class B1 receptors support the notion that the N-terminal α-helix (broadly the ECD region) is involved in regulating receptor-mediated signal transduction in addition to its constitutive role of binding to the peptide C terminus. Obviously, the physiological significance of these observations require in-depth investigations including the role of ECD in basal activities of certain class B1 receptors. Together with 14 other class B1 GPCRs having full-length structures, the addition of the PACAP27-bound VIP2R–G_s_ complex structure will allow us to perform a class-wide analysis and comparison of their structural and functional features with a goal of developing better therapeutic agents for a variety of human diseases.

## Methods

### Construct

To build the VIP2R(24–438) construct, human VIP2R (residues 24–438) was cloned into pFastBac (Invitrogen) with an N-terminal Flag tag followed by a 8×His tag. A TEV protease cleavage site followed by a double MBP (2MBP) tag and LgBiT at the C terminus via homologous recombination (CloneExpress One Step Cloning Kit, Vazyme). The native signal peptide was replaced with the prolactin precursor sequence to increase the protein expression. The VIP2R(1–438) construct is similar to VIP2R(24–438) but without any modification at the N terminus. The resultant VIP2R(24–438) and VIP2R(1–438) constructs retained the full-length receptor sequence (residues 24–438 excluding the signal peptide sequence and residues 1–438, respectively), different from that of VIP1R^13^ and PAC1R^14,15^ where either C-terminal truncation, mutations or their combination were made. A dominant-negative bovine Gα_s_ (DNGα_s_) construct was generated by site-directed mutagenesis to incorporate mutations S54N, G226A, E268A, N271K, K274D, R280K, T284D, I285T, and A366S to decrease the affinity of nucleotide-binding and increase the stability of Gαβγ complex^30^. Rat Gβ1 was cloned with an N-terminal 6×His tag and a C-terminal HiBiT connected with a 15-residue linker. All three G protein components together with bovine Gγ2 were cloned into a pFastBac vector, respectively. Sequences of all primers used in this study are provided in Supplementary Table 6.

### Cell culture

*Spodoptera* *frugiperda* (*Sf*9) insect cells (lnvitrogen) were cultured in ESF 921 serum-free medium (Expression Systems) at 27 °C and 120 rpm. Cell cultures were grown to a density of 2.5 × 10^6^ cells mL^−1^ and then infected with baculoviruses expressing VIP2R–LgBiT fusion, DNGα_s_, Gβ1–HiBiT fusion and Gγ2, respectively, at the ratio of 1:2:2:2. The cells were collected by centrifugation at 813 × *g* for 20 min after infection for 48 h, and kept frozen at −80 °C until use.

### PACAP27–VIP2R–G_s_ complex formation and purification

Cell pellets from 1 L culture were thawed and lysed in the lysis buffer (20 mM HEPES, pH 7.4, 100 mM NaCl, 10% (v/v) glycerol). The complex formation was initiated by the addition of 10 μM PACAP27 (Synpeptide), 20 μg/mL Nb35, 25 mU/mL apyrase (NEB), 5 mM CaCl_2_, 5 mM MgCl_2_, and 250 μM TCEP, supplemented with EDTA-free protease inhibitor cocktail (Bimake) for 1.5 h incubation at room temperature (RT). The membrane was solubilized by 0.5% (w/v) lauryl maltose neopentyl glycol (LMNG; Anatrace) and 0.1% (w/v) cholesterol hemisuccinate (CHS; Anatrace) for 2 h at 4 °C. After centrifugation at 65,000 × *g* for 40 min, the supernatant was separated and incubated with amylose resin (NEB) for 2 h at 4 °C. The resin was collected and packed into a gravity flow column and washed with 20 column volumes of 5 μM PACAP27, 0.01% (w/v) LMNG, 0.002% (w/v) CHS, 0.01% (w/v) GDN, 0.008% (w/v) CHS, 20 mM HEPES, pH 7.4, 100 mM NaCl, 10% (v/v) glycerol, 2 mM MgCl_2_, 2 mM CaCl_2_, and 25 μM TCEP. 2MBP-tag was removed by His-tagged TEV protease (customer-made) during overnight incubation. The complex was concentrated using an Amicon Ultra Centrifugal filter (MWCO, 100 kDa) and subjected to a Superose 6 Increase 10/300 GL column (GE Healthcare) that was pre-equilibrated with running buffer containing 20 mM HEPES, pH 7.4, 100 mM NaCl, 2 mM MgCl_2_, 2 mM CaCl_2_, 250 μM TCEP, 5 μM PACAP27, 0.00075% (w/v) LMNG, 0.00025% (w/v) GDN, 0.00025% digitonin, and 0.0002% (w/v) CHS. Eluted fractions containing the PACAP27**–**VIP2R**–**G_s_ complex were pooled and concentrated. All procedures mentioned above were performed at 4 °C.

### Expression and purification of Nb35

The nanobody 35 (Nb35) with a C-terminal 6×His tag was expressed in *E. coli* BL21 (DE3) bacteria and cultured in TB medium supplemented with 2 mM MgCl_2_, 0.1% (w/v) glucose, and 50 μg/mL ampicillin to an OD600 value of 0.7–1.2 at 37 °C. The culture was then induced by 1 mM IPTG and grown overnight incubation at 28 °C. Cells were harvested by centrifugation (1626 × *g*, 20 min) and Nb35 protein was extracted and purified by nickel affinity chromatography as previously described^31^. Eluted protein was concentrated and subjected to a HiLoad 16/600 Superdex 75 column (GE Healthcare) pre-equilibrated with buffer containing 20 mM HEPES, pH 7.5, and 100 mM NaCl. The monomeric fractions supplemented with 30% (v/v) glycerol were flash frozen in liquid nitrogen and stored at −80 °C until use.

### Cryo-EM data acquisition

The purified PACAP27**–**N-terminal modified VIP2R(24-438)**–**G_s_ complex (3 μL at 3.7 mg per mL) and PACAP27**–**VIP2R(1-438)**–**G_s_ complex (3 μL at 8.5 mg per mL) were applied to glow-discharged holey carbon grids (Quantifoil R1.2/1.3). Vitrification was performed using a Vitrobot Mark IV (ThermoFisher Scientific) at 100% humidity and 4 °C. Cryo-EM images were processed on a Titan Krios microscope (FEI) equipped with a Gatan K3 Summit direct electron detector and serial EM3.7 were used to acquire cryo-EM images. The microscope was operated at 300 kV accelerating voltage, at a nominal magnification of 46,685× in counting mode, corresponding to a pixel size of 1.071 Å. In total, 4753 movies of the PACAP27–VIP2R(24-438)–G_s_ complex and 8130 movies of the PACAP27–VIP2R(1-438)–G_s_ complex were obtained with a defocus range of −1.2 to −2.2 μm. An accumulated dose of 80 electrons per Å^2^ was fractionated into a movie stack of 36 frames.

Dose-fractionated image stacks were subjected to beam-induced motion correction using MotionCor2.1. A sum of all frames, filtered according to the exposure dose, in each image stack was used for further processing. Contrast transfer function parameters for each micrograph were determined by Gctf v1.06. For the PACAP27–N-terminal modified VIP2R(24-438)–G_s_ complex, particle selection, 2D, and 3D classifications were performed on a binned dataset with a pixel size of 2.142 Å using RELION-3.1.1. Auto-picking yielded 5,558,869 particle projections that were subjected to reference-free 2D classification to discard false-positive particles or particles categorized in poorly defined classes, producing 1,729,374 particle projections for further processing. This subset of particle projections was subjected to a round of maximum-likelihood-based 3D classifications with a pixel size of 2.142 Å, resulting in one well-defined subset with 931,248 projections. Further 3D classifications with a mask on the receptor produced one good subset accounting for 602,466 particles, which were subjected to another round of 3D classifications with a mask on the ECD. A selected subset containing 305,004 projections was then subjected to 3D refinement and Bayesian polishing with a pixel size of 1.071 Å. After the last round of refinement, the final map has an indicated global resolution of 3.4 Å at a Fourier shell correlation (FSC) of 0.143. Local resolution was determined using the Bsoft package (v2.0.3) with half maps as input maps.

For the PACAP27–VIP2R(1-438)–G_s_ complex, particle selection, 2D and 3D classifications were performed on a binned dataset with a pixel size of 2.142 Å using RELION-3.1.1. Auto-picking yielded 10,130,817 particle projections that were subjected to reference-free 2D classification to discard false-positive particles or particles categorized in poorly defined classes, producing 2,353,199 particle projections for further processing. This subset of particle projections was subjected to a round of maximum-likelihood-based 3D classifications with a pixel size of 2.142 Å, resulting in one well-defined subset with 1,751,675 projections. Further 3D classifications with a mask on the receptor produced one good subset accounting for 1,018,806 particles, which were subjected to another round of 3D classifications with a mask on the ECD. A selected subset containing 770,771 projections was then subjected to 3D refinement and Bayesian polishing with a pixel size of 1.071 Å. After the last round of refinement, the final map has an indicated global resolution of 2.7 Å at a Fourier shell correlation (FSC) of 0.143. Local resolution was determined using the Bsoft package (v2.0.3) with half maps as input maps.

### Model building and refinement

The model of the PACAP27–VIP2R–G_s_ complex was built using the cryo-EM structure of the PACAP27–VIP1R–G_s_ complex (PDB code: 6VN7) and the crystal structure of VIP2R ECD (PDB code: 2X57) as the starting point. The model was docked into the EM density map using UCSF Chimera v1.13.1^32^, followed by iterative manual adjustment and rebuilding in COOT 0.9.4.1^33^. Real space refinement was performed using Phenix v1.16^34^. The model statistics were validated using MolProbity v4.2^35^. Structural figures were prepared in UCSF Chimera v1.13.1, UCSF ChimeraX v1.0, and PyMOL v.2.1 (https://pymol.org/2/). The final refinement statistics are provided in Supplementary Table 1.

### Molecular dynamics simulations

Molecular dynamic simulations were performed by Gromacs 2020.1. The peptide–VIP2R complexes were built based on the cryo-EM structure of the PACAP27–VIP2R(1-438)–G_s_ complex and prepared by the Protein Preparation Wizard (Schrödinger 2017-4) with the G protein and Nb35 nanobody removed. The receptor chain termini were capped with acetyl and methyl amide. All titratable residues were left in their dominant state at pH 7.0 by PROPKA included in Schrödinger 2017-4. To build MD simulation systems, the complexes were embedded in a bilayer composed of 287 POPC lipids and solvated with 0.15 M NaCl in explicit TIP3P waters using CHARMM-GUI Membrane Builder v3.5^36^. The CHARMM36-CAMP force filed^37^ was adopted for protein, peptides, lipids, and salt ions. The Particle Mesh Ewald (PME) method was used to treat all electrostatic interactions beyond a cut-off of 12 Å and the bonds involving hydrogen atoms were constrained using LINCS algorithm^38^. The complex system was first relaxed using the steepest descent energy minimization, followed by slow heating of the system to 310 K with restraints. The restraints were reduced gradually over 50 ns. Finally, 1000 ns restrain-free production run was carried out for each simulation, with a time step of 2 fs in the NPT ensemble at 310 K and 1 bar using the Nose-Hoover thermostat and the semi-isotropic Parrinello-Rahman barostat^39^, respectively. Each system was replicated to perform three independent simulations. The interface area was calculated by the program FreeSASA 2.0^40^, using the Sharke-Rupley algorithm with a probe radius of 1.2 Å. Similar simulation procedure and analysis were adopted for the MD simulations of PACAP27-bound N-terminal modified VIP2R by Flag and 8×His tags, which were modeled to the cryo-EM structure of PACAP27–N-terminal modified VIP2R(24-438)–G_s_ complex using BioLuminate and Prime (Schrödinger 2017-4). The starting configuration of the MD model and all necessary input files are provided as Supplementary Data 1.

### cAMP accumulation assay

WT or mutant VIP2Rs, VIP1Rs, and PAC1Rs were cloned into pcDNA3.1 vector (Invitrogen) for functional studies. CHO-K1 cells were transiently transfected with the vectors using Lipofectamine 2000 transfection reagent (Invitrogen) and incubated at 37 °C in 5% CO_2_. After 24 h, the transfected cells were digested with 0.02% (w/v) EDTA, resuspended in stimulation buffer (Hanks’ balanced salt solution (HBSS) supplemented with 5 mM HEPES, 0.5 mM IBMX and 0.1% (w/v) BSA, pH 7.4) to a density of 0.6 million cells per mL and added to 384-well white plates (3000 cells per well). cAMP accumulation was measured by a LANCE Ultra cAMP kit (PerkinElmer) according to the manufacturer’s instructions. In brief, transfected cells were incubated for 40 min in stimulation buffer with different concentrations of ligand (5 μL) at RT. The reaction was stopped by the addition of a lysis buffer containing 5 μL Eu-cAMP tracer and 5 μL ULight-anti-cAMP. Plates were then incubated for 60 min at RT and time-resolved FRET signals were measured at 620 and 665 nm, respectively, by an EnVision multilabel plate reader (PerkinElmer). Data were analyzed in GraphPad Prism 8.3 and converted to absolute cAMP levels using a standard curve. The E_max_ data were normalized to the WT (E_max_ of WT was defined as 100%).

### Whole cell binding assay

CHO-K1 cells were cultured in F12 medium with 10% FBS and seeded at a density of 30,000 cells/well in Isoplate-96 plates (PerkinElmer). Twenty-four hours after transfection with the WT or mutant receptors, CHO-K1 cells were washed twice and incubated with blocking buffer (F12 supplemented with 25 mM HEPES and 0.1% (w/v) BSA, pH 7.4) for 2 h at 37 °C. For homogeneous competition binding, radiolabeled ^125^I-PACAP27 (40 pM, PerkinElmer) and seven decreasing concentrations of unlabeled peptides were added separately and competitively reacted with the cells in blocking buffer at RT for 3 h. Following incubation, cells were washed three times with ice-cold PBS and lysed by 50 μL lysis buffer (PBS supplemented with 20 mM Tris-HCl, 1% Triton X-100, pH 7.4). The radioactivity was subsequently counted (counts per minute, CPM) in a scintillation counter (MicroBeta2 Plate Counter, PerkinElmer) using a scintillation cocktail (OptiPhase SuperMix, PerkinElmer). The span data were normalized to the WT (span of WT was defined as 100%).

### Receptor surface expression

Cell surface expression was determined by flow cytometry to the N-terminal Flag tag on the WT VIP2R(1-23-Flag-VIP2R(24–438)) and its mutants transiently expressed in CHO-K1 cells. All the mutant constructs were modified by single-point mutation in the setting of the WT construct. Briefly, approximately 3 × 10^5^ cells were blocked with PBS containing 5% BSA (w/v) at RT for 15 min, and incubated with 1:300 anti-Flag primary antibody (diluted with PBS containing 5% BSA, Sigma-Aldrich) at RT for 1 h. The cells were then washed three times with PBS containing 1% BSA (w/v) followed by 1 h incubation with 1:1000 anti-mouse Alexa Fluor 488 conjugated secondary antibody (diluted with PBS containing 5% BSA, Invitrogen) at 4 °C in the dark. After washing three times, cells were re-suspended in 200 μL PBS containing 1% BSA for detection by Flow Cytometer (BD Biosciences) utilizing laser excitation and emission wavelengths of 488 and 530 nm, respectively. For each sample, 10,000 cellular events were collected, and the total fluorescence intensity of positive expression cell population was calculated by NovoExpress 1.2.1. Data were normalized to the WT receptor.

### Statistical analysis

All functional data were presented as means ± standard error of the mean (S.E.M.). Statistical analysis was performed using GraphPad Prism 8.3 (GraphPad Software). Concentration-response curves were evaluated with a three-parameter logistic equation. The significance was determined by one-way ANOVA with Dunnett’s multiple comparison test. Significant difference is accepted at *P* < 0.05.

### Reporting summary

Further information on research design is available in the Nature Research Reporting Summary linked to this article.

## Supplementary information

Supplementary Information

Description of Additional Supplementary Files

Supplementary Data 1

Reporting Summary

## Data Availability

All relevant data are available from the corresponding authors upon reasonable request. The raw data underlying Figs. 1b, 3f, 4b, Supplementary Figs. 1d–e, 1g–h, 1j–m, 2d, 2h and Supplementary Tables 3–5 are provided as a Source Data file. The starting configuration of the MD model and all necessary input files are provided as Supplementary Data 1. The atomic coordinates and electron microscopy maps have been deposited in the Protein Data Bank (PDB) under accession codes: 7VQX (PACAP27–VIP2R(1-438)–G_s_) and 7WBJ (PACAP27–N-terminal modified VIP2R(24-438)–G_s_), and Electron Microscopy Data Bank (EMDB) accession codes: EMD-32095 (PACAP27–VIP2R(1-438)–G_s_) and EMD-32401 (PACAP27–N-terminal modified VIP2R(24-438)–G_s_), respectively. Source data are provided with this paper.

## Source data

Source Data
