# Supplementary material for: A distinctive ligand recognition mechanism by the human vasoactive intestinal polypeptide receptor 2
Source: Nat Commun. 2022 Apr 27;13:2272. doi: 10.1038/s41467-022-30041-z (PMC9046186; doi:10.1038/s41467-022-30041-z)
Supplement: Supplementary file 1 — Supplementary Information [file 41467_2022_30041_MOESM1_ESM.pdf]

Supplementary Information

A distinctive ligand recognition mechanism by the human vasoactive intestinal polypeptide receptor 2

Brief description of what this file includes:

Supplementary Fig. 1 | Purification and characterization of the PACAP27–VIP2R–G_s–Nb35 complex.

Supplementary Fig. 2 | Cryo-EM analysis of the PACAP27–VIP2R–G_s complex.

Supplementary Fig. 3 | Atomic resolution models of the PACAP27–N-terminal modified VIP2R(24-438)–G_s and PACAP27–VIP2R(1-438)–G_s complexes in the cryo-EM density maps.

Supplementary Fig. 4 | Molecular dynamics (MD) simulations of PACAP27-bound active VIP2R.

Supplementary Fig. 5 | Gating strategy of the cell surface expression assay.

Supplementary Table 1 | Cryo-EM data collection, refinement and validation statistics.

Supplementary Table 2 | Interaction between PACAP27 and VIP2R in the PACAP27–VIP2R(1-438)–G_s complex structure (PDB code: 7VQX).

Supplementary Table 3 | Effects of residue mutation in the ligand-binding pocket on PACAP27-induced cAMP responses and cell surface expression at VIP2R, VIP1R or PAC1R mutants.

Supplementary Table 4 | Receptor binding profiles of PACAP27 at VIP2R, VIP1R and PAC1R.

Supplementary Table 5 | Effects of VIP2R, VIP1R or PAC1R N terminus modification on PACAP27-induced cAMP responses, ligand binding profiles and cell surface expression.

Supplementary Table 6 | Primers used in this study, related to Figures 3, 4, Supplementary Figure 1 and Supplementary Tables 3-5.

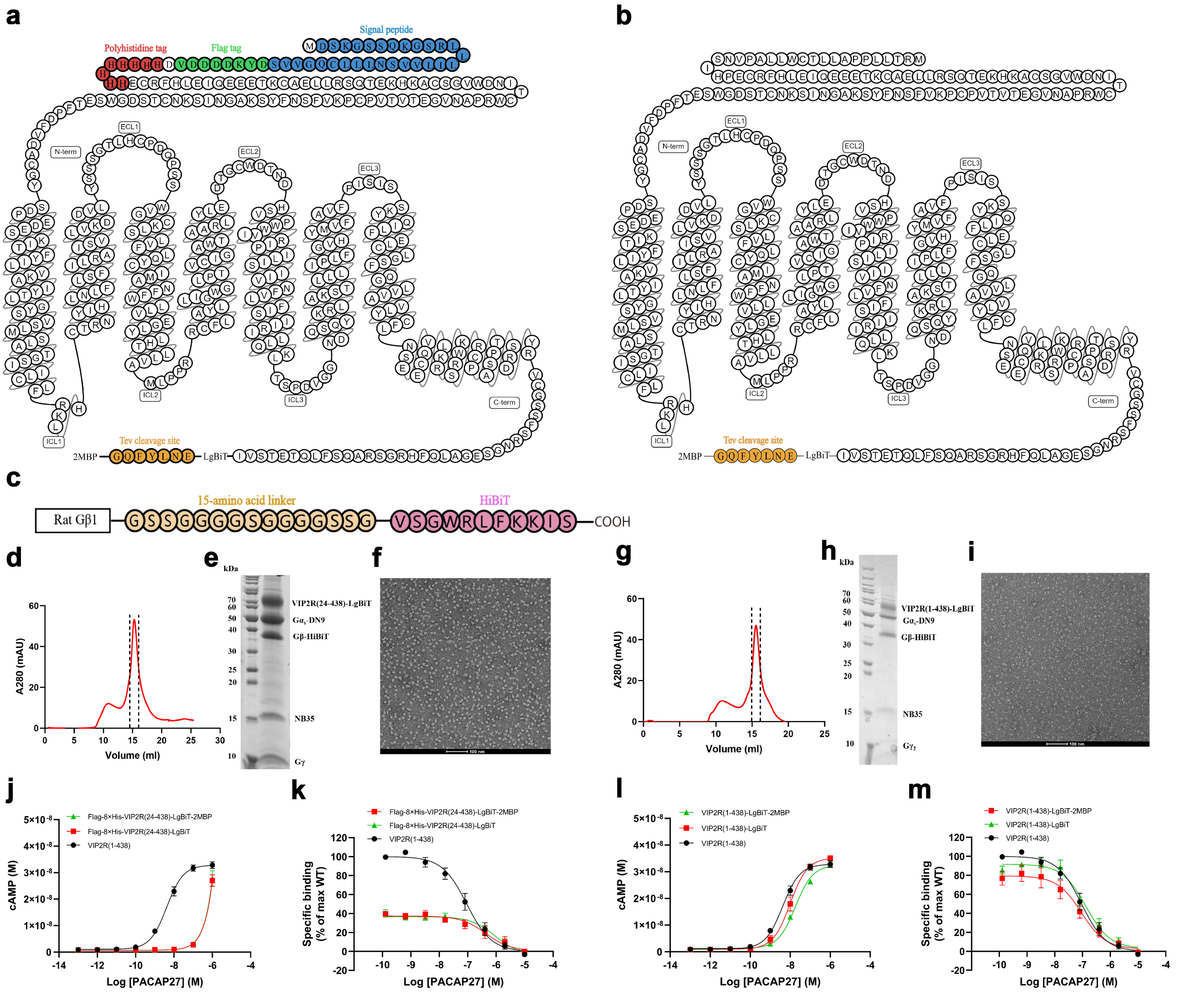

Supplementary Figure 1. Purification and characterization of the PACAP27–VIP2R–G_s–Nb35 complex. **a**, Snake-plot diagram of the human VIP2R–LgBiT construct Flag–8×His–VIP2R(24-438)–LgBiT–2MBP. The resultant PACAP27–N-terminal modified VIP2R(24-438)–G_s complex structure was determined by cryo-EM at 3.4 Å. **b**, Snake-plot diagram of the human VIP2R(1-438)–LgBiT–2MBP. The resultant PACAP27–VIP2R(1-438)–G_s complex structure was determined by cryo-EM at 2.7 Å. **c**, Gβ1 constructs used for structure determination. Rat Gβ1 was attached to HiBiT with a 15-amino acid (15AA) linker between them. **d-f**, Analytical size-exclusion chromatography of the PACAP27–N-terminal modified VIP2R(24-438)–G_s (**d**), SDS-PAGE/Coomassie blue stain (**e**) and representative negative staining image (**f**) of the purified PACAP27–N-terminal modified VIP2R(24-438)–G_s complex. **g-i**, Analytical size-exclusion chromatography of the PACAP27–VIP2R(1-438)–G_s complex (**g**), SDS-PAGE/Coomassie blue stain (**h**) and representative negative staining image (**i**) of the purified PACAP27–VIP2R(1-438)–G_s complex. These experiments (**d-i**) were repeated independently three times with similar results. **j**, cAMP responses following PACAP27 stimulation in CHO-K1

cells transfected with the N-terminal modified VIP2R(24-438). **k**, Binding of PACAP27 to the N-terminal modified VIP2R(24-438) in competition with ^{125}I-PACAP27$_{1-27}$. **l**, cAMP responses following PACAP27 stimulation in CHO-K1 cells transfected with wild-type (WT) VIP2R(1-438). **m,** Binding of PACAP27 to the WT VIP2R(1-438) in competition with ^{125}I-PACAP27$_{1-27}$. All data were generated and graphed as means ± S.E.M. of at least three independent experiments (*n*=3-6).

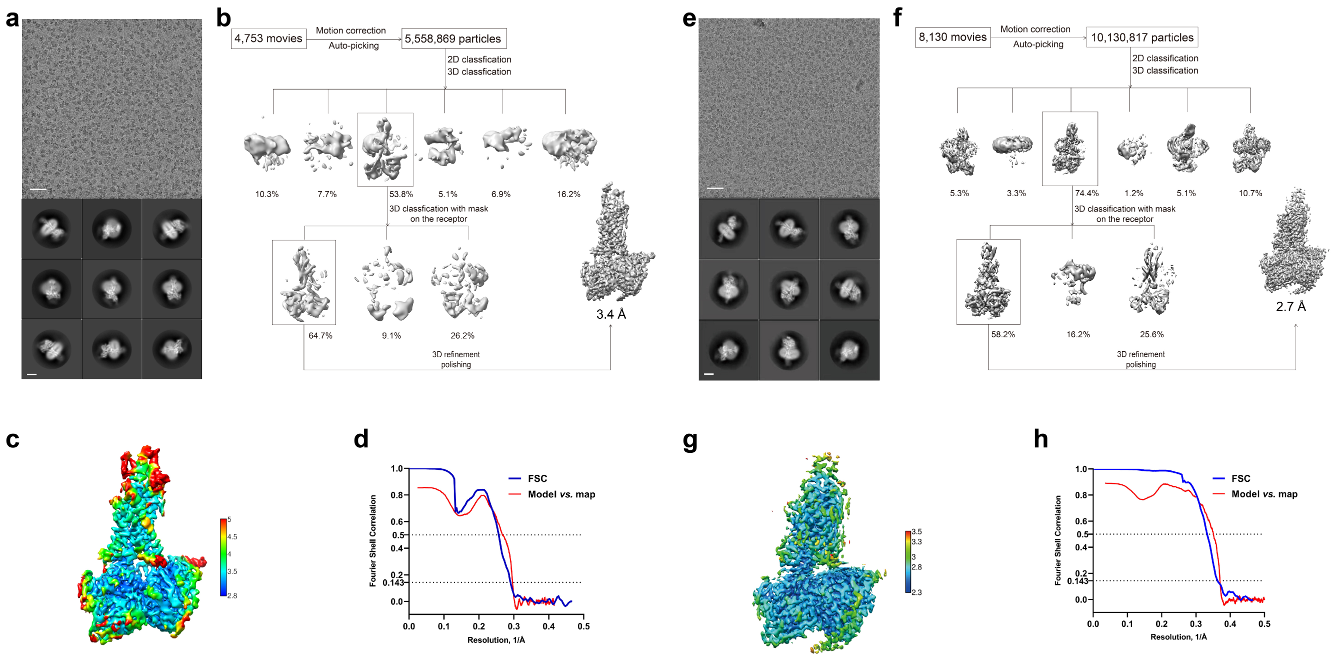

Supplementary Figure 2. Cryo-EM analysis of the PACAP27–VIP2R–G$_s$ complex. a-d, PACAP27–N-terminal modified VIP2R(24-438)–G$_s$ complex. **a**, representative cryo-EM micrograph (scale bar: 40 nm) and two-dimensional class averages (scale bar: 5 nm). **b**, flowchart of cryo-EM data processing. **c**, local resolution distribution map. **d**, Gold-standard Fourier shell correlation (FSC) curves of the map (blue) and the model *vs*. map (red). **e-h,** PACAP27–VIP2R(1-438)–G$_s$ complex. **e**, representative cryo-EM micrograph (scale bar: 40 nm) and two-dimensional class averages (scale bar: 5 nm). **f**, flowchart of cryo-EM data processing. **g**, local resolution distribution map **h**, Gold-standard Fourier shell correlation (FSC) curves of the map (blue) and the model *vs*. map (red). Source data are provided as a Source Data file.

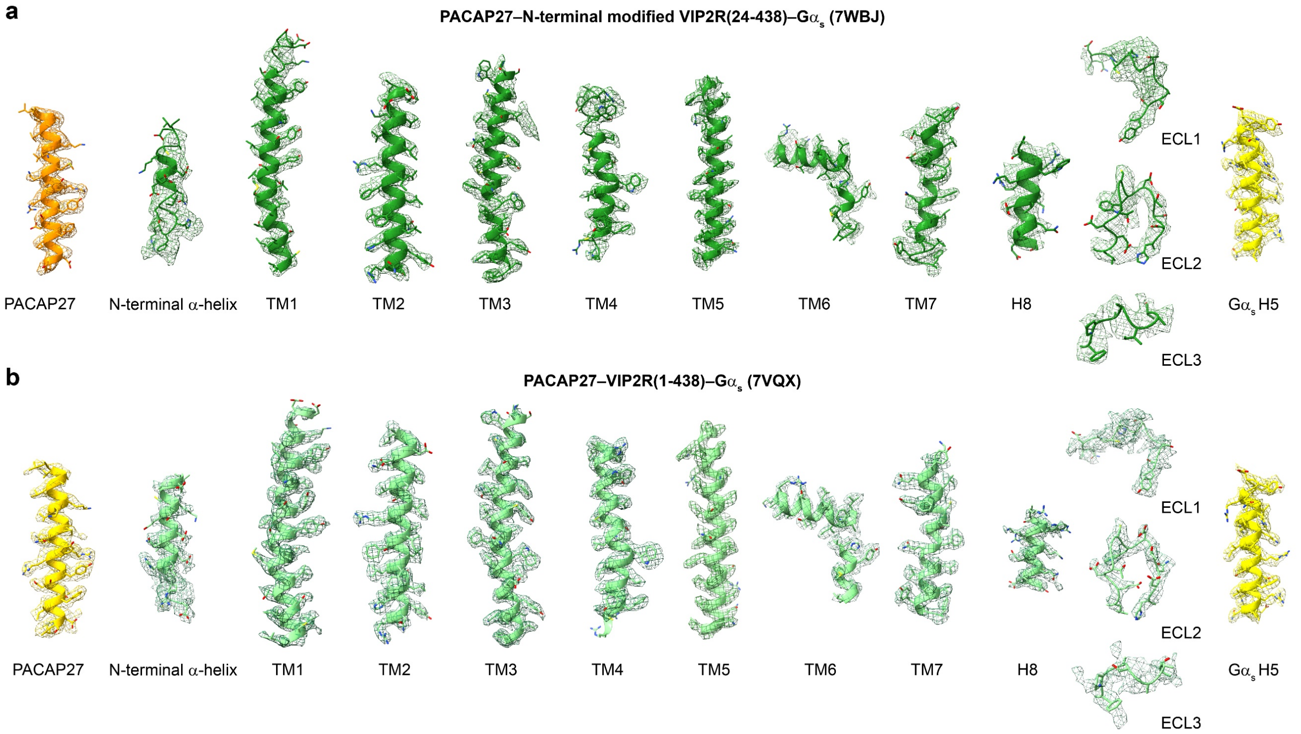

Supplementary Figure 3. Atomic resolution models of the PACAP27–N-terminal modified VIP2R(24-438)–G_s and PACAP27–VIP2R(1-438)–G_s complexes in the cryo-EM density maps. a, EM density map and model of the PACAP27–N-terminal modified VIP2R(24-438)–G_s are shown for all seven transmembrane α-helices, helix 8, N-terminal α-helix and all extracellular loops of VIP2R(24-438), the α5-helix (H5) of the $G\alpha_s$ Ras-like domain and PACAP27. **b,** EM density map and model of the PACAP27–VIP2R(1-438)–G_s are shown for all seven transmembrane α-helices, helix 8, N-terminal α-helix and all extracellular loops of VIP2R(1-438), the α5-helix of the $G\alpha_s$ Ras-like domain and PACAP27.

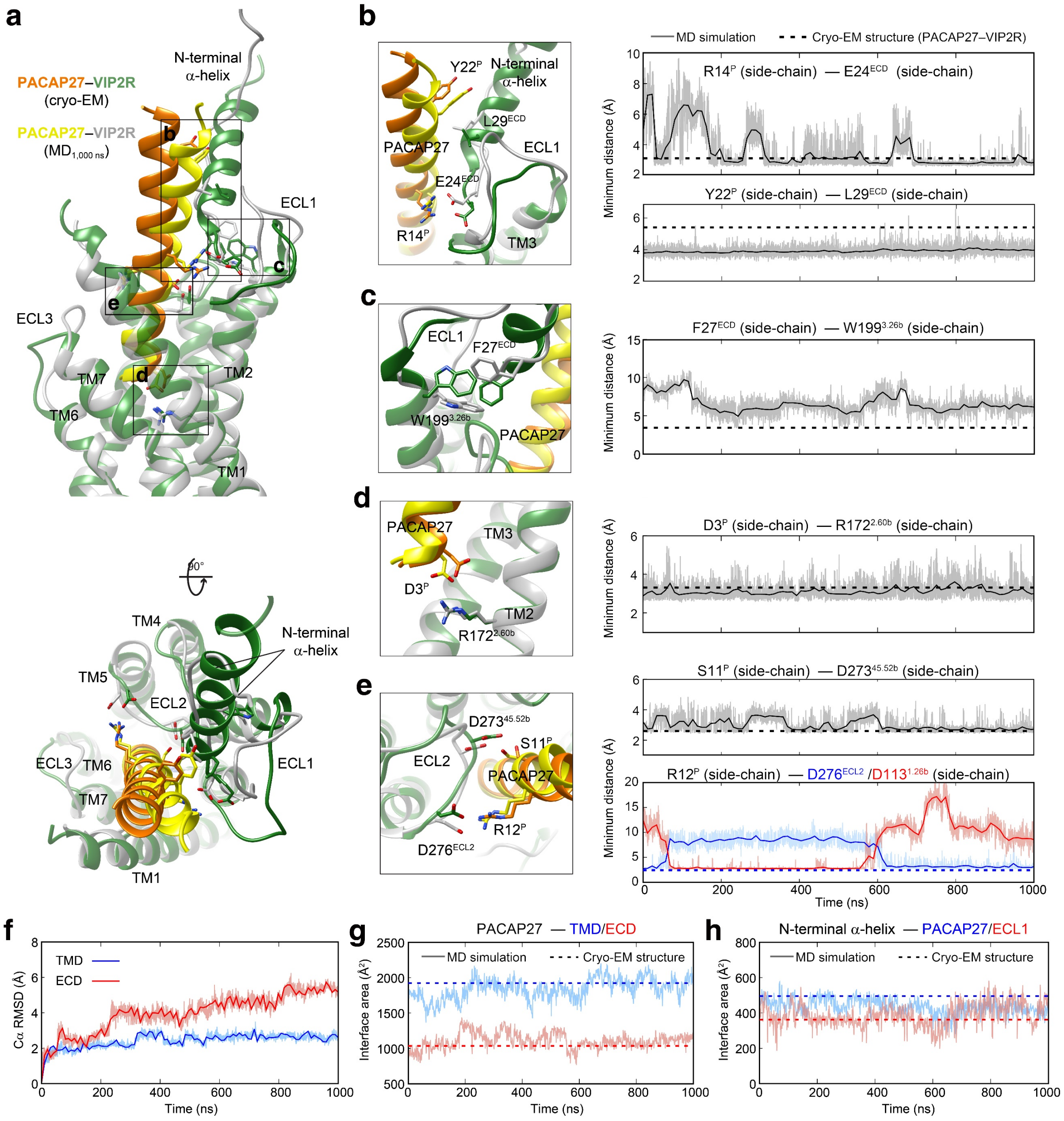

Supplementary Figure 4. Molecular dynamics (MD) simulations of PACAP27-bound active VIP2R. a, Comparison of the N-terminal α-helix conformation between the final simulation snapshot at 1,000 ns and the cryo-EM structure of PACAP27–VIP2R(1-438)–G_s complex (PDB code: 7VQX). G protein and VIP2R ECD except the N-terminal α-helix are omitted for clarity. The key residues in the peptide-receptor interface are shown in sticks. **b,** A close-up view of the interactions among two peptide-N-terminal α-helix pairs and their minimum distances during MD simulations. **c,** A close-up view of the interaction between $F27^{ECD}$ and $W199^{3.26b}$ and the minimum distance during MD simulations. **d-e,** Close-up views of the interactions among three peptide-receptor pairs and their minimum distances during MD simulations. **f,** Root mean square deviation (RMSD) of Cα positions of the VIP2R ECD and TMD, where all snapshots were superimposed on the cryo-EM structure

of VIP2R ECD and TMD using the Cα atoms, respectively. **g,** The buried surface area between PACAP27 and ECD (red) or TMD (blue), calculated by freeSASA 2.0. **h,** The buried surface area between the N-terminal α-helix of VIP2R ECD and ECL1 (red) or PACAP27 (blue), calculated by freeSASA 2.0. The thick and thin traces represent moving averages and original, unsmoothed values obtained from one single MD simulation trajectory, respectively. The MD simulations were repeated independently three times with similar results.

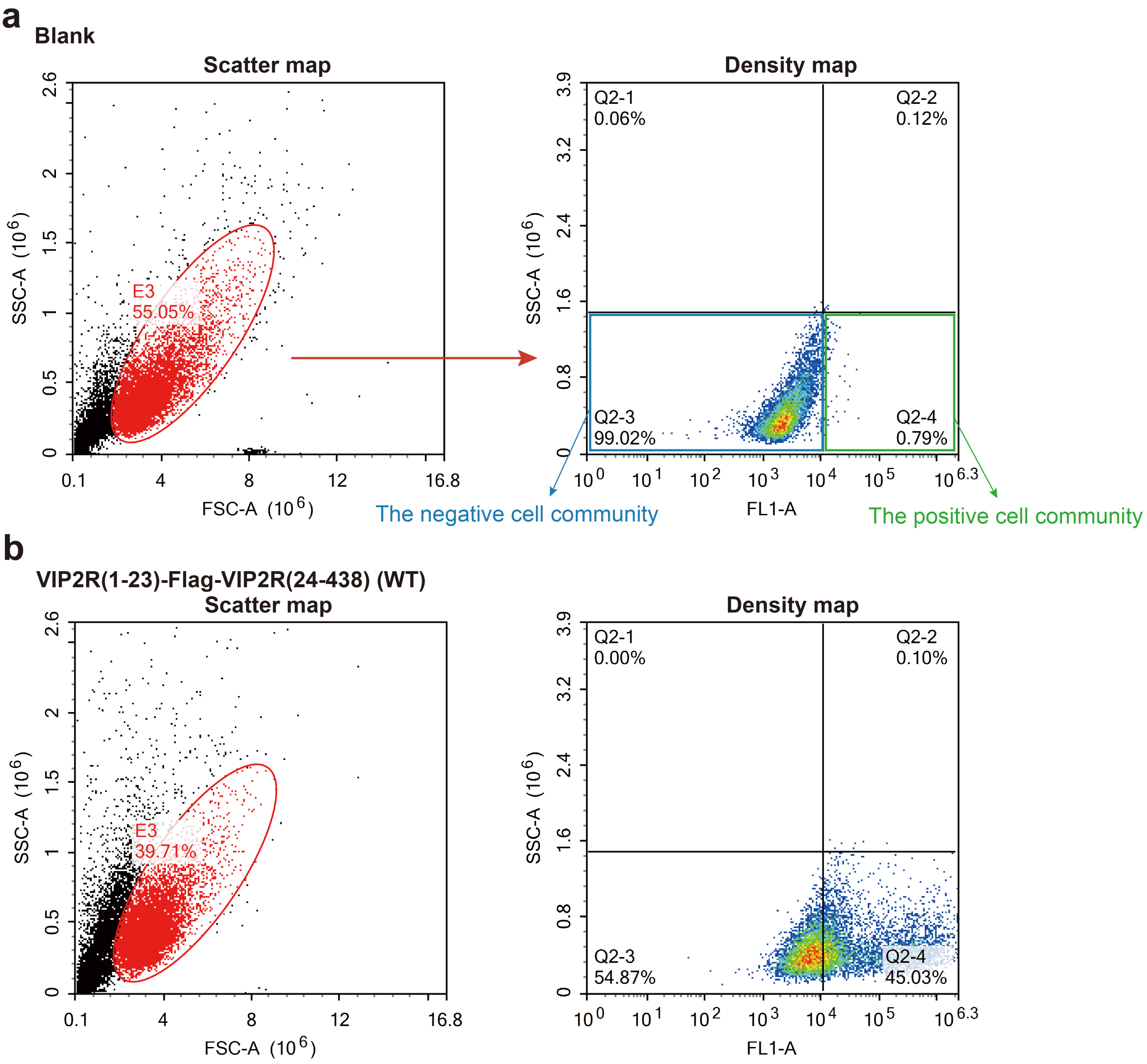

Supplementary Figure 5. Gating strategy of the cell surface expression assay. Circle a gate E3 in the scatter map (red circle) and the cells shown in the density map are all the cells in the gate E3 of the scatter map. Fluorescence signal intensity (FITC) is presented by density map. With the blank sample (no receptor transfection) as the reference value of background fluorescence signal (**a**), the "quadrant gate" divides the fluorescence signal density map into four quadrants. The third quadrant represents the negative cell community, while the fourth quadrant represents the positive cell community. The expression value of wild-type (WT) receptor (**b**) can be calculated as follows: (M(Q2-4)-M(Q2-3)) × (Q2-4% Parent). The calculation of receptor mutants is the same as that of the WT receptor, which was then normalized with the WT receptor to calculate the relative expression value of the mutants.

Supplementary Table 1 | Cryo-EM data collection, refinement and validation statistics.

	**PACAP27–N-terminal modified VIP2R(24-438)–G$_s$**	**PACAP27–VIP2R(1-438)–G$_s$**
**Data collection and processing**		
Magnification	46,685	46,685
Voltage (kV)	300	300
Electron exposure (e^{-}/Å^2)	80	80
Defocus range (μm)	-1.2 to -2.2	-1.2 to -2.2
Pixel size (Å)	1.071	1.071
Symmetry imposed	Cl	Cl
Initial particle images (no.)	5,558,869	10,130,817
Final particle images (no.)	305,004	770,771
Map resolution (Å)	3.42	2.74
FSC threshold	0.143	0.143
Map resolution range (Å)	3.1 – 5.0	2.3 – 4.3
**Refinement**		
Initial model used (PDB code)	PDB codes 6VN7 and 2X57	PDB codes 6VN7 and 2X57
Model resolution (Å)	3.98	2.93
FSC threshold	0.5	0.5
Model resolution range (Å)	3.2 – 5.2	2.5 – 3.8
Map sharpening B factor (Å^2)	-168.8	-120.6
Model composition		
Non-hydrogen atoms	8,986	9,049
Protein residues	1,146	1,148
B factors (Å^2)		
Protein	69.03	49.93
Ligand	73.37	59.23
R.m.s. deviations		
Bond lengths (Å)	0.004	0.004
Bond angles (°)	0.607	0.547
Validation		
MolProbity score	1.93	1.68
Clash score	11.86	8.08
Poor rotamers (%)	0	0
Ramachandran plot		
Favored (%)	95.12	96.37
Allowed (%)	4.88	3.63
Disallowed (%)	0	0

Supplementary Table 2 | Interaction between PACAP27 and VIP2R in the PACAP27–VIP2R(1-438)–G$_s$ complex structure (PDB code: 7VQX).

**PACAP27**	**VIP2R**
H1^{P}	Hydrogen bond with Q210$^{3.37b}$ (side-chain)<br>Hydrophobic contacts with I213$^{3.40b}$ and M214$^{3.41b}$
S2^{P}	Hydrogen bond with E360$^{7.42b}$ (side-chain)
D3^{P}	Salt bridge with R172$^{2.60b}$ (side-chain)<br>Hydrogen bond with Y134$^{1.47b}$ (side-chain)
G4^{P}	
I5^{P}	Hydrophobic contacts with W281$^{5.36b}$ and I357$^{7.39b}$
F6^{P}	Stacking with Y130$^{1.43b}$<br>Hydrophobic contacts with Y123$^{1.36b}$, V126$^{1.39b}$, K127$^{1.40b}$, Y130$^{1.43b}$, I357$^{7.39b}$ and L361$^{7.43b}$
T7^{P}	Hydrogen bond with K179$^{2.67b}$ (side-chain)
D8^{P}	Hydrogen bond with N275^{ECL2} (backbone)
S9^{P}	Hydrogen bond with Y123$^{1.36b}$ (side-chain)
Y10^{P}	Stacking with Y123$^{1.36b}$ and Y184^{ECL1}<br>Hydrophobic contacts with K127$^{1.40b}$
S11^{P}	Hydrogen bonds with D273$^{45.52b}$ (side-chain) and Y184^{ECL1} (side-chain)
R12^{P}	Salt bridge with D276^{ECL2} (side-chain)<br>Hydrogen bond with N275^{ECL2} (backbone)
Y13^{P}	Hydrogen bond with D116$^{1.29b}$ (backbone)<br>Hydrophobic contacts with K119$^{1.32b}$ and I120$^{1.33b}$
R14^{P}	Salt bridge with E24^{ECD} (side-chain)<br>Stacking with Y184^{ECL1}<br>Hydrophobic contacts with C25^{ECD}
K15^{P}	Hydrogen bond with V78^{ECD} (backbone)<br>Hydrophobic contacts with F82^{ECD}
Q16^{P}	Hydrogen bond with D113^{ECD} (side-chain)
M17^{P}	
A18^{P}	
V19^{P}	Hydrophobic contacts with F79^{ECD} and F82^{ECD}
K20^{P}	Salt bridges with D116$^{1.29b}$ (side-chain) and E117$^{1.30b}$ (side-chain)<br>Hydrophobic contacts with Y111^{ECD}
K21^{P}	
Y22^{P}	Hydrogen bond with Q32^{ECD} (side-chain)<br>Hydrophobic contacts with I59^{ECD}
L23^{P}	Hydrophobic contacts with I59^{ECD}, F79^{ECD} and F105^{ECD}
A24^{P}	
A25^{P}	
V26^{P}	Hydrophobic contacts with I59^{ECD}
L27^{P}	Hydrophobic contacts with I59^{ECD}, F105^{ECD} and V106^{ECD},

Supplementary Table 3 | Effects of residue mutation in the ligand-binding pocket on PACAP27-induced cAMP responses and cell surface expression at VIP2R, VIP1R or PAC1R mutants.

Receptor	Mutation	cAMP accumulation		Cell surface expression (% WT)
		pEC_{50} ± S.E.M.	E_{max} ± S.E.M. (% WT)	
	WT	8.38 ± 0.06	100.00	100.00
	R26^{ECD}A	8.70 ± 0.15	96.07 ± 12.63	66.20 ± 7.84
	F27^{ECD}A	7.35 ± 0.15***	117.72 ± 17.98	122.23 ± 16.45
	H28^{ECD}A	8.30 ± 0.18	106.33 ± 23.12	100.27 ± 18.08
	R26^{ECD}A+F27^{ECD}A+H28^{ECD}A	7.26 ± 0.04 ***	101.37 ± 6.06	60.12 ± 4.67
	F79^{ECD}A	6.40 ± 0.11***	106.55 ± 12.02	109.16 ± 7.90
	Y111^{ECD}A	7.98 ± 0.10	123.20 ± 10.14	100.92 ± 9.15
	D116$^{1.29b}$A+ E117$^{1.30b}$A	7.67 ± 0.09***	120.45 ± 6.30	67.46 ± 9.29
	K119$^{1.32b}$A	8.48 ± 0.09	90.65 ± 5.90	122.63 ± 14.55
VIP2R	Y123$^{1.36b}$ A	6.19 ± 0.22***	102.51 ± 17.55	102.78 ± 8.87
	Y184^{ECL1}A	6.36 ± 0.13***	109.80 ± 11.77	120.10 ± 20.31
	D194^{ECL1}A	8.13 ± 0.11	111.69 ± 13.21	82.37 ± 2.11
	W199$^{3.26b}$A	8.03 ± 0.12	114.08 ± 18.77	77.51 ± 11.65
	L209$^{3.36b}$F	8.91 ± 0.03**	112.01 ± 1.18	95.25 ± 2.84
	N275^{ECL2}A	7.97 ± 0.06	109.08 ± 6.33	53.30 ± 9.81
	D276^{ECL2}A	8.15 ± 0.09	112.29 ± 14.38	69.11 ± 6.25
	S353$^{7.35b}$K	7.89 ± 0.09*	104.18 ± 11.56	132.61 ± 14.87
	Q356$^{7.38b}$A	7.78 ± 0.10**	112.87 ± 8.02	93.10 ± 11.45
	I357$^{7.39b}$A	6.57 ± 0.11***	116.41 ± 14.51	122.43 ± 5.93
	WT	9.70 ± 0.11	100.00	100.00
	Q135$^{1.32b}$A	9.69 ± 0.04	116.49 ± 0.18	107.73 ± 11.72
	Y139$^{1.36b}$A	7.67 ± 0.14***	105.43 ± 19.27	98.96 ± 6.84
	F222$^{3.36b}$L	9.66 ± 0.05	120.39 ± 3.05	115.57 ± 15.87
VIP1R	I289^{ECL2}A	8.14 ± 0.10***	93.06 ± 12.43	94.29 ± 8.75
	N290$^{5.32b}$A	9.68 ± 0.06	115.90 ± 7.71	112.29 ± 11.65
	P366$^{7.35b}$K	9.63 ± 0.04	123.40 ± 0.65	97.38 ± 11.40
	K369$^{7.38b}$A	8.35 ± 0.23***	97.87 ± 17.34	94.87 ± 9.12
	M370$^{7.39b}$A	8.83 ± 0.11***	55.62 ± 6.07**	115.80 ± 8.78
	WT	9.63 ± 0.11	100.00	100.00
	Q146$^{1.34b}$A	9.94 ± 0.06	116.07 ± 4.98	103.68 ± 7.14
	Y150$^{1.36b}$A	8.04 ± 0.35***	100.85 ± 13.88	103.46 ± 7.44
	F233$^{3.36b}$L	9.82 ± 0.04	116.43 ± 0.65	89.39 ± 9.43
PAC1R	N300^{ECL2}A	8.96 ± 0.09*	55.54 ± 3.33***	94.16 ± 10.11
	N301^{ECL2}A	9.39 ± 0.09	55.87 ± 4.16***	92.87 ± 12.55
	K378$^{7.35b}$S	8.89 ± 0.19**	102.04 ± 15.57	106.71 ± 2.82
	R381$^{7.38b}$A	8.31 ± 0.10***	105.70 ± 9.60	90.63 ± 10.83
	L382$^{7.39b}$A	8.63 ± 0.10***	53.79 ± 3.18***	87.56 ± 15.74

cAMP production data were converted to absolute cAMP levels using a standard curve and concentration-response curves were analyzed using a three-parameter logistic equation to obtain pEC_{50} values. The experiments were carried out independently at least three times with similar results (*n*=3-6). Cell surface expression was assessed by FACS. Values were normalized to the wild-type (WT, shown as percentage) in CHO-K1 cells. All the mutant constructs were modified by single-point mutation in the setting of the WT construct. Data shown are means ± S.E.M. from three independent experiments (*n*=3). Source data are provided as a Source Data file. One-way ANOVA with Dunnett's multiple comparison test was used to determine statistical difference (*P< 0.05, **P< 0.01, ***P< 0.001).

Supplementary Table 4 | Receptor binding profiles of PACAP27 at VIP2R, VIP1R and PAC1R.

**Receptor**	**Mutation**	**pIC$_{50}$ ± S.E.M.**	**Span ± S.E.M. (% WT)**
VIP2R	WT	6.78 ± 0.08	100.00
	F27^{ECD}A	6.49 ± 0.25	49.28 ± 6.31***
	R26^{ECD}A+F27^{ECD}A+H28^{ECD}A	6.03 ± 0.16	35.64 ± 2.05***
	F79^{ECD}A	6.07 ± 0.32	30.21 ± 2.95***
	Y111^{ECD}A	7.14 ± 0.21	87.48 ± 9.24
	D116$^{1.29b}$A+ E117$^{1.30b}$A	6.98 ± 0.21	62.47 ± 6.83**
	K119$^{1.32b}$A	7.25 ± 0.19	88.80 ± 7.53
	Y123$^{1.36b}$ A	6.34 ± 0.37	32.30 ± 4.72***
	Y184^{ECL1}A	6.28 ± 0.34	33.44 ± 5.94***
	W199$^{3.26b}$A	6.93 ± 0.20	67.80 ± 4.80*
	L209$^{3.36b}$F	6.97 ± 0.17	95.07 ± 5.40
	N275^{ECL2}A	7.08 ± 0.22	74.56 ± 11.46
	S353$^{7.35b}$K	6.79 ± 0.23	72.14 ± 12.88
	Q356$^{7.38b}$A	6.82 ± 0.26	77.83 ± 18.17*
	I357$^{7.39b}$A	6.48 ± 0.23	59.72 ± 6.19***
VIP1R	WT	7.27 ± 0.06	100.00
	Y139$^{1.36b}$A	7.09 ± 0.21	30.15 ± 6.03***
	I289^{ECL2}A	7.59 ± 0.18	56.18 ± 9.03***
	K369$^{7.38b}$A	7.48 ± 0.13	56.67 ± 1.53***
	M370$^{7.39b}$A	7.75 ± 0.13*	55.78 ± 8.78***
PAC1R	WT	6.68 ± 0.07	100.00
	Y150$^{1.36b}$A	6.65 ± 0.29	64.22 ± 14.75*
	N300^{ECL2}A	6.29 ± 0.11	84.94 ± 9.71
	K378$^{7.35b}$S	6.77 ± 0.19	74.51 ± 3.33
	R381$^{7.38b}$A	6.45 ± 0.20	58.39 ± 19.91*
	L382$^{7.39b}$A	6.36 ± 0.25	47.04 ± 12.04**

Whole cell binding assay was performed in CHO-K1 cells. Binding data were analyzed using a three-parameter logistic equation to determine pIC$_{50}$ and span values. Data shown are means ± S.E.M. from at least three independent experiments (*n*=3-8). Source data are provided as a Source Data file. One-way ANOVA with Dunnett's multiple comparison test was used to determine statistical difference (*P< 0.05, **P< 0.01, ***P< 0.001). WT, wild-type.

Supplementary Table 5 | Effects of VIP2R, VIP1R or PAC1R N terminus modification on PACAP27-induced cAMP responses, ligand binding profiles and cell surface expression.

Receptor	N terminus modification	cAMP accumulation		Ligand binding		Cell surface expression (% WT)
		pEC_{50} ± S.E.M.	E_{max} ± S.E.M. (% WT)	pIC_{50} ± S.E.M.	Span ± S.E.M. (% WT)	
VIP2R	WT	8.38 ± 0.06	100.00	6.78 ± 0.06	100.00	100.00
	VIP2R-Δ2	8.33 ± 0.15	71.49 ± 11.68**	5.84 ± 0.47	76.84 ± 14.01	115.43 ± 6.11
	VIP2R-Δ5	ND	ND	ND	ND	69.68 ± 8.37
	VIP2R-Δ10	ND	ND	ND	ND	49.97 ± 1.78*
	VIP2R+$[AA]_2$	6.08 ± 0.17***	ND	6.01 ± 0.46	54.96 ± 22.74*	138.20 ± 12.85
	VIP2R+$[AA]_5$	5.84 ± 0.33***	ND	5.89 ± 0.48	60.35 ± 25.06	189.07 ± 20.09***
	VIP2R+$[AA]_{10}$	5.71 ± 0.31***	ND	5.77 ± 0.39*	48.32 ± 13.05*	107.20 ± 11.43
	PAC1R(1-138)-VIP2R(113-438)	6.97 ± 0.05***	101.27 ± 5.23	6.05 ± 0.11	38.91 ± 2.75**	54.30 ± 5.03
	VIP2R(1-23)-PAC1R(21-41)-VIP2R(45-438)	ND	ND	6.16 ± 0.16	35.50 ± 3.90**	148.83 ± 13.89*
VIP1R	WT	9.70 ± 0.11	100.00	7.27 ± 0.06	100.00	100.00
	VIP1R-Δ2	9.64 ± 0.04	122.73 ± 0.69***	7.56 ± 0.16	111.01 ± 17.63	62.31 ± 11.92
	VIP1R-Δ5	9.62 ± 0.04	122.90 ± 0.83***	7.39 ± 0.23	113.58 ± 34.22	61.89 ± 7.86
	VIP1R-Δ10	6.75 ± 0.09***	51.70 ± 4.25***	ND	ND	209.47 ± 21.19***
	VIP1R-ΔECD	ND	ND	ND	ND	39.33 ± 5.66**
	VIP1R+$[AA]_2$	9.63 ± 0.04	123.09 ± 1.78***	7.53 ± 0.22	119.00 ± 39.94	136.22 ± 8.32
	VIP1R+$[AA]_5$	9.62 ± 0.04	123.24 ± 0.96***	7.38 ± 0.15	111.03 ± 20.96	121.25 ± 7.34
	VIP1R+$[AA]_{10}$	9.63 ± 0.06	118.71 ± 6.82**	7.62 ± 0.16*	119.60 ± 27.56	111.02 ± 17.09
PAC1R	WT	9.63 ± 0.11	100.00	6.68 ± 0.07	100.00	100.00
	PAC1R-Δ2	10.04 ± 0.10*	109.90 ± 10.14	6.74 ± 0.24	110.20 ± 26.58	82.24 ± 10.45
	PAC1R -Δ5	8.98 ± 0.08**	53.85 ± 2.41***	6.83 ± 0.21	73.40 ± 19.80*	92.73 ± 11.75
	PAC1R -Δ10	7.39 ± 0.13***	50.63 ± 5.25***	ND	ND	102.25 ± 8.58
	PAC1R -ΔECD	ND	ND	ND	ND	41.90 ± 6.34**
	PAC1R+$[AA]_2$	9.89 ± 0.11	109.04 ± 12.40	6.61 ± 0.25	107.14 ± 28.61	118.87 ± 16.35
	PAC1R+$[AA]_5$	9.85 ± 0.07	107.52 ± 5.76	6.77 ± 0.23	115.00 ± 24.83	168.96 ± 11.61***
	PAC1R+$[AA]_{10}$	9.99 ± 0.20	86.87 ± 21.38	6.65 ± 0.14	121.16 ± 20.07	142.94 ± 7.34*

cAMP production data were converted to absolute cAMP levels using a standard curve and concentration-response curves were analyzed using a three-parameter logistic equation to obtain pEC_{50} values. Data shown are means ± S.E.M. from at least three independent experiments (*n*=3-6). Whole cell binding assay was performed in CHO-K1 cells. Binding data were analyzed using a three-parameter logistic equation to determine pIC_{50} and span values (*n*=3-8). Cell surface expression was assessed by FACS. Values were normalized to the wild-type (WT, shown as percentage) in CHO-K1 cells. Data shown are means ± S.E.M. from three independent

experiments (*n*=3). Source data are provided as a Source Data file. One-way ANOVA with Dunnett's multiple comparison test was used to determine statistical difference (*P< 0.05, **P< 0.01, ***P< 0.001). ND, values that could not be determined due to incomplete curve fits. Neither receptor binding nor cAMP activity was observed in N terminus truncations beyond 10 amino acids of VIP2R (Δ15 and Δ20).

Supplementary Table 6 | Primers used in this study, related to Figures 3, 4, Supplementary Figure 1 and Supplementary Tables 3-5.

Oligonucleotide name	Oligonucleotide sequence (5'-3')	Cloning method	Product
Insert-LgBiT-forward	AAACGGAGACCTCGGTCATCGTCTTCACACTCGAAGATTTCGTTG	Homologous recombination	pcDNA3.1-VIP2R(1-438)-LgBiT
Insert-LgBiT-reverse	GGGTTTAAACGGGCCCTCTATTAGCTGTTGATGGTTACTCGGAA		
Linear-pcDNA3.1-forward-1	TAGAGGGCCCGTTTAAACCC		
Linear-pcDNA3.1-reverse-1	GATGACCGAGGTCTCCGTTTG		
Insert-LgBiT-2MBP-forward	AAACGGAGACCTCGGTCATCGTCTTCACACTCGAAGATTTCGTTG	Homologous recombination	pcDNA3.1-VIP2R(1-438)-LgBiT-2MBP
Insert-LgBiT-2MBP-reverse	GGGTTTAAACGGGCCCTCTATTACTTGGTGATACGAGTCTGCGC		
Linear-pcDNA3.1-forward-2	TAGAGGGCCCGTTTAAACCC		
Linear-pcDNA3.1-reverse-2	GATGACCGAGGTCTCCGTTTG		
Insert-PAC1R(21-41)-forward	CCGTGAACAGCATTCACCCAATGCATTCTGACTGCATCTTCAA	Homologous recombination	pcDNA3.1-VIP2R(1-23)-PAC1R(21-41)-VIP2R(45-438)
Insert-PAC1R(21-41)-reverse	GCTTTGTGTTTTTCTGTTTGGGCCCTCTGGATCTTCTCCA		
Linear-pcDNA3.1-forward-3	CAAACAGAAAAACACAAAGCCTGC		
Linear-pcDNA3.1-reverse-3	TGGGTGAATGCTGTTCACGG		
Insert-PAC1R(1-138)-forward	GAGACCCAAGCTGGCTAGCGATGGCTGGTGTCGTGCACG	Homologous recombination	pcDNA3.1-PAC1R(1-138)-VIP2R(113-438)
Insert-PAC1R(1-138)-reverse	TTGCTCTCATCCTCCGGGTCTTCATCAAACCCACAGGCATC		
Linear-pcDNA3.1-forward-4	GACCCGGAGGATGAGAGCA		
Linear-pcDNA3.1-reverse-4	CGCTAGCCAGCTTGGGTCT		
R26A-forward	CCAGAATGCGCATTTCATCTGGAAATACAGGAGGAA	Site-directed mutagenesis	pcDNA3.1-VIP2R(1-438)-Q26A
R26A-reverse	TGAAATGCGCATTCTGGGTGAATGCTGTTCAC		
F27A-forward	AATGCCGAGCTCATCTGGAAATACAGGAGGAAGAAA		pcDNA3.1-VIP2R(1-438)-F27A
F27A-reverse	CAGATGAGCTCGGCATTCTGGGTGAATGCTGT		
H28A-forward	GAATGCCGATTTGCTCTGGAAATA		pcDNA3.1-VIP2R(1-438)-H28A

H28A-reverse	TATTTCCAGAGCAAATCGGCATTC		
R26A +F27A+H28A-forward	GCAGCTGCTCTGGAAATACAGGAGGAAGAAACAA		pcDNA3.1-VIP2R(1-438)-R26A +F27A+H28A
R26A +F27A+H28A-reverse	ATTTCCAGAGCAGCTGCGCATTCTGGGTGAATGCTGTTC		
F79A-forward	CAAAAGTCGCCAGCAATTTTTACAGCAAAGCAGG		pcDNA3.1-VIP2R(1-438)-F79A
F79A-reverse	ATTGCTGGCGACTTTTGGGCAGGGCACCGTGA		
Y111A-forward	ATGCCTGTGGCGCCAGCGACCCGGAGGATGAG		pcDNA3.1-VIP2R(1-438)-Y111A
Y111A-reverse	GCTGGCGCCACAGGCATCGACGAAATCTGGGA		
D116A+E117A-forward	AGGCTGCGAGCAAGATCACGTTTTATATTCTGGT		pcDNA3.1-VIP2R(1-438)-D116A+E117A
D116A+E117A-reverse	TGATCTTGCTCGCAGCCTCCGGGTCGCTGTAGCC		
K119A-forward	AGAGCGCGATCACGTTTTATATTCTGGTGAAGGC		pcDNA3.1-VIP2R(1-438)-K119A
K119A-reverse	AAACGTGATCGCGCTCTCATCCTCCGGGTCGC		
Y123A-forward	CACGTTTGCTATTCTGGTGAAGGCCATTTATACA		pcDNA3.1-VIP2R(1-438)-Y123A
Y123A-reverse	CCAGAATAGCAAACGTGATCTTGCTCTCATCCTC		
Y184A-forward	TTCTCGCCTCCAGCTCTGGCACGTTGCACTGC		pcDNA3.1-VIP2R(1-438)-Y184A
Y184A-reverse	AGAGCTGGAGGCGAGAACGTCGTCCTTGACCAGC		
D194A-forward	ACTGCCCTGCCCAGCCATCCT		pcDNA3.1-VIP2R(1-438)-D194A
D194A-reverse	AGGATGGCTGGGCAGGGCAGT		
W199A-forward	ATCCTCCGCGGTGGGCTGCAAGCTGAGCCTGG		pcDNA3.1-VIP2R(1-438)-W199A
W199A-reverse	AGCCCACCGCGGAGGATGGCTGGTCAGGGCAG		
L209F-forward	TGGTCTTCTTTCAGTACTGCA		pcDNA3.1-VIP2R(1-438)-L209F
L209F-reverse	TGCAGTACTGAAAGAAGACCA		
N275A-forward	TGGGATACAGCCGACCACAGT		pcDNA3.1-VIP2R(1-438)-N275A
N275A-reverse	ACTGTGGTCGGCTGTATCCCA		

D276A-forward	ATACAAACGCCCACAGTGTGC		pcDNA3.1-VIP2R(1-438)-D276A
D276A-reverse	GCACACTGTGGGCGTTTGTAT		
S353K-forward	CCATCAGCATCTCCAAAAAATACCAGATACTGTTTGAGCTGTGC		pcDNA3.1-VIP2R(1-438)-S353K
S353K-reverse	TTTGGAGATGCTGATGGGAAACACGGCAAACA		
Q356A-forward	CCAAATACGCGATACTGTTTGAGCTGTGCCTCGG		pcDNA3.1-VIP2R(1-438)-Q356A
Q356A-reverse	CAGTATCGCGTATTTGGAGGAGATGCTGATGG		
I357A-forward	ATACCAGGCACTGTTTGAGCTGTGCCTCGGGT		pcDNA3.1-VIP2R(1-438)-I357A
I357A-reverse	CAAACAGTGCCTGGTATTTGGAGGAGATGCTGA		
VIP2R-Δ2-forward	GCATTCACCCACGATTTCATCTGGAAATACAGGAG		VIP2R(1-23)-(26-438)
VIP2R-Δ2-reverse	GAAATCGTGGGTGAATGCTGTTCACGGGGGCGA		
VIP2R-Δ5-forward	GCATTCACCCACTGGAAATACAGGAGGAAGAAACAA		VIP2R(1-23)-(29-438)
VIP2R-Δ5-reverse	TTTCCAGTGGGTGAATGCTGTTCACGGGGGCGA		
VIP2R-Δ10-forward	CAGCATTCACCCAGAAGAAACAAAATGTGCAGAGCTT		VIP2R(1-23)-(34-438)
VIP2R-Δ10-reverse	TCTTCTGGGTGAATGCTGTTCACGGGGGCGAGC		
VIP2R+[AA]$_2$-forward	TTCACCCAGGCTCGGAATGCCGATTTCATCTGGAA		VIP2R(1-23)+[AA]$_2$-(24-438)
VIP2R+[AA]$_2$-reverse	ATTCCGAGCCTGGGTGAATGCTGTTCACGGGGG		
VIP2R+[AA]$_5$-forward	AGGCTCGAGCGGTGGTGAATGCCGATTTCATCTGGAA		VIP2R(1-23)+[AA]$_5$-(24-438)
VIP2R+[AA]$_5$-reverse	CACCACCGCTCGAGCCTGGGTGAATGCTGTTCACGG		
VIP2R+[AA]$_{10}$-forward	AGCGGTGGTGGCGGGAGCGGAGGTGAATGCCGATTTCATCTGGAA		VIP2R(1-23)+[AA]$_{10}$-(24-438)
VIP2R+[AA]$_{10}$-reverse	GCTCCCGCCACCACCGCTCGAGCCTGGGTGAATGCTGTTCACGG		
Q135A-forward	CAGGCGACCATGTTCTACGGTTCTGTGAAGAC	Site-directed mutagenesis	pcDNA3.1-VIP1R(1-457)-Q135A
Q135A-reverse	TAGAACATGGTCGCCTGCTCATCCAAACTCGCTGC		
Y139A-forward	ATGTTCGCCGGTTCTGTGAAGACCGGCTACAC		pcDNA3.1-VIP1R(1-457)-Q139A

Y139A-reverse	ACAGAACCGGCGAACATGGTCTGCTGCTCATCC		
F222L-forward	GGTCTTTTTACAATATTGTGT		pcDNA3.1-VIP1R(1-457)-F222L
F222L-reverse	ACACAATATTGTAAAAAGACC		
I289A-forward	TGGGACACCGCCAACTCCTCA		pcDNA3.1-VIP1R(1-457)-I289A
I289A-reverse	TGAGGAGTTGGCGGTGTCCCA		
N290A-forward	ACACCATCGCCTCCTCACTGTGGTGGATCATAAAG		pcDNA3.1-VIP1R(1-457)-N290A
N290A-reverse	TGAGGAGGCGATGGTGTCCCAGCACCCATAAT		
P366K-forward	GAAGGAAGTGAAGATGGTCTTTGAGCTCGTCG		pcDNA3.1-VIP1R(1-457)-P366K
P366K-reverse	CCATCTTCACTTCCTTCTTAAAATTGTCCGGAAAGAAGGC		
K369A-forward	TGAAGTGGCGATGGTCTTTGAGCTCGTCGTGG		pcDNA3.1-VIP1R(1-457)-K369A
K369A-reverse	AGACCATCGCCACTTCAGGCTTAAAATTGTCCG		
M370A-forward	AAGTGAAGGCGGTCTTTGAGC		pcDNA3.1-VIP1R(1-457)-M370A
M370A-reverse	GCTCAAAGACCGCCTTCACTT		
VIP1R-Δ2-forward	GCTGCAGGAGGAGTGTGACTATGTGCAGATGAT		VIP1R(1-30)-(33-457)
VIP1R-Δ2-reverse	TCACACTCCTCCTGCAGCGCCTGGCCGCCCGCCGG		
VIP1R-Δ5-forward	GGAGTGTGACTATGTGCAGATGATCGAGGTGCA		VIP1R(1-30)-(36-457)
VIP1R-Δ5-reverse	TGCACATAGTCACACTCCGCCTGGCCGCCCGCCGG		
VIP1R-Δ10-forward	GCAGATGATCGAGGTGCAGCACAAGCAGTGCCT		VIP1R(1-30)-(41-457)
VIP1R-Δ10-reverse	TGCACCTCGATCATCTGCGCCTGGCCGCCCGCCGG		
VIP1R-ΔECD-forward	GGCGGCCAGGCGGCGAGTTTGGATGAGCAGCAG		VIP1R(1-30)-(129-457)
VIP1R-ΔECD-reverse	ATCCAAACTCGCCGCCTGGCCGCCCGCCGGCCC		
VIP1R+[AA]$_2$-forward	GGGCTCGGCCAGGCTGCAGGAGGAGTGTGACTA		VIP1R(1-30)+[AA]$_2$-(31-457)
VIP1R+[AA]$_2$-reverse	TGCAGCCTGGCCGAGCCCGCCTGGCCGCCCGCCGG		

VIP1R+[AA]$_5$-forward	GGGCTCGAGCGGTGGTGCCAGGCTGCAGGAGGAG		VIP1R(1-30)+[AA]$_5$-(31-457)
VIP1R+[AA]$_5$-reverse	CACCACCGCTCGAGCCCGCCTGGCCGCCCGCCGG		
VIP1R+[AA]$_{10}$-forward	AGCGGTGGTGGCGGGAGCGGAGGTGCCAGGCTGCAGGAGGAG		VIP1R(1-30)+[AA]$_{10}$-(31-457)
VIP1R+[AA]$_{10}$-reverse	GCTCCCGCCACCACCGCTCGAGCCCGCCTGGCCGCCCGCCGG		
Q146A-forward	TGGGGACGCGGATTATTACTACCTGTCAGTGAAGGCC	Site-directed mutagenesis	pcDNA3.1-PAC1R(1-468)-Q146A
Q146A-reverse	AATAATCCGCGTCCCCAGTCTCAGATTCATATTCA		
Y150A-forward	TTATTACGCCCTGTCAGTGAAGGCCCTCTACAC		pcDNA3.1-PAC1R(1-468)-Y150A
Y150A-reverse	CTGACAGGGCGTAATAATCCTGGTCCCCAGTCTC		
F233L-forward	CGTCATGGTTTTCTTACACTACTGTGTTGTGTCCAACTACTTCT		pcDNA3.1-PAC1R(1-468)-F233L
F233L-reverse	GTAAGAAAACCATGACGGCCTTACATTCCACA		
N300A-forward	GGATATGGCTGACAGCACAGCTCTGTGGTGGG		pcDNA3.1-PAC1R(1-468)-N300A
N300A-reverse	TGCTGTCAGCCATATCCCAGCAGCCTGTGTCA		
D301A-forward	TATGAATGCCAGCACAGCTCTGTGGTGGGTGA		pcDNA3.1-PAC1R(1-468)-D301A
D301A-reverse	CTGTGCTGGCATTCATATCCCAGCAGCCTGTG		
K378S-forward	TGTCAGCAGCAGGGAAAGACTCGTGTTTGAGC		pcDNA3.1-PAC1R(1-468)-K378S
K378S-reverse	TTTCCCTGCTGCTGACATTCTCTGGGGAGAAG		
R381A-forward	AAGGGAAGCACTCGTGTTTGAGCTGGGGCTGG		pcDNA3.1-PAC1R(1-468)-R381A
R381A-reverse	ACACGAGTGCTTCCCTTTTGCTGACATTCTCTG		
L382A-forward	GGAAAGAGCCGTGTTTGAGCTGGGGCTGGGCT		pcDNA3.1-PAC1R(1-468)-L382A
L382A-reverse	CAAACACGGCTCTTTCCCTTTTGCTGACATTCT		
PAC1R-Δ2-forward	TGCCTCTGACTGCATCTTCAAGAAGGAGCAAGC		PAC1R(1-20)-(23-468)
PAC1R-Δ2-reverse	AAGATGCAGTCAGAGGCAGGGGCCATAGGCAGC		
PAC1R-Δ5-forward	CCCTGCCATCTTCAAGAAGGAGCAAGCCATGTG		PAC1R(1-20)-(26-468)

PAC1R-Δ5-reverse	TTCTTGAAGATGGCAGGGGCCATAGGCAGCAGG		
PAC1R-Δ10-forward	TGCCCAAGCCATGTGCCTGGAGAAGATCCAGAG		PAC1R(1-20)-(31-468)
PAC1R-Δ10-reverse	AGGCACATGGCTTGGGCAGGGGCCATAGGCAGC		
PAC1R-ΔECD-forward	ATGGCCCCTGCCTATGAATCTGAGACTGGGGAC		PAC1R(1-20)-(139-468)
PAC1R-ΔECD-reverse	CTCAGATTCATAGGCAGGGGCCATAGGCAGCAG		
PAC1R+$[AA]_2$-forward	CGGCTCGATGCATTCTGACTGCATCTTCAAGAA		PAC1R(1-20)+$[AA]_2$-(21-468)
PAC1R+$[AA]_2$-reverse	TCAGAATGCATCGAGCCGGCAGGGGCCATAGGCAG		
PAC1R+$[AA]_5$-forward	GGCTCGAGCGGTGGTATGCATTCTGACTGCATCTTCAA		PAC1R(1-20)+$[AA]_5$-(21-468)
PAC1R+$[AA]_5$-reverse	ATACCACCGCTCGAGCCGGCAGGGGCCATAGGCAG		
PAC1R+$[AA]_{10}$-forward	AGCGGTGGTGGCGGGAGCGGAGGTATGCATTCTGACTGCATCTTCAA		PAC1R(1-20)+$[AA]_{10}$-(21-468)
PAC1R+$[AA]_{10}$-reverse	GCTCCCGCCACCACCGCTCGAGCCGGCAGGGGCCATAGGCAG		
