# Supplementary material for: A distinctive ligand recognition mechanism by the human vasoactive intestinal polypeptide receptor 2
Source: Nat Commun. 2022 Apr 27;13:2272. doi: 10.1038/s41467-022-30041-z (PMC9046186; doi:10.1038/s41467-022-30041-z)
Supplement: Supplementary file 2 — Description of Additional Supplementary Files [file 41467_2022_30041_MOESM2_ESM.pdf]

File name: Supplementary Data 1

Description: The starting configuration and the input files of the MD simulations/ model.
